# Non-Pharmacological Self-Management Strategies for Chemotherapy-Induced Peripheral Neuropathy in People with Advanced Cancer: A Systematic Review and Meta-Analysis

**DOI:** 10.3390/nu14122403

**Published:** 2022-06-09

**Authors:** Megan Crichton, Patsy M. Yates, Oluwaseyifunmi Andi Agbejule, Amy Spooner, Raymond J. Chan, Nicolas H. Hart

**Affiliations:** 1 Cancer and Palliative Care Outcomes Centre, School of Nursing, Queensland University of Technology, Brisbane, QLD 4059, Australia; p.yates@qut.edu.au (P.M.Y.); a.spooner@qut.edu.au (A.S.); raymond.chan@flinders.edu.au (R.J.C.); nicolas.hart@flinders.edu.au (N.H.H.); 2 Caring Futures Institute, College of Nursing and Health Science, Flinders University, Adelaide, SA 5042, Australia; andi.agbejule@flinders.edu.au; 3 Exercise Medicine Research Institute, School of Medical and Health Sciences, Edith Cowan University, Perth, WA 6027, Australia; 4 Institute for Health Research, The University of Notre Dame Australia, Perth, WA 6959, Australia

**Keywords:** chemotherapy-induced peripheral neuropathy, CIPN, neuropathy, chemotherapy, advanced cancer, hematology

## Abstract

Non-pharmacological self-management interventions for chemotherapy-induced peripheral neurotherapy (CIPN) are of clinical interest; however, no systematic review has synthesized the evidence for their use in people with advanced cancer. Five databases were searched from inception to February 2022 for randomized controlled trials assessing the effect of non-pharmacological self-management interventions in people with advanced cancer on the incidence and severity of CIPN symptoms and related outcomes compared to any control condition. Data were pooled with meta-analysis. Quality of evidence was appraised using the Revised Cochrane Risk of Bias Tool for Randomized Trials (RoB2), with data synthesized narratively. Grading of Recommendations, Assessment, Development and Evaluations (GRADE) was applied to assess the certainty of the evidence. Thirteen studies were included, which had a high (69%) or unclear (31%) risk of bias. Greatest confidence was found for physical exercise decreasing CIPN severity (SMD: −0.89, 95% CI: −1.37 to −0.41; *p* = 0.0003; *I*^2^ = 0%; *n* = 2 studies, *n* = 76 participants; GRADE level: moderate) and increasing physical function (SMD: 0.51, 95% CI: 0.02 to 1.00; *p* = 0.04; *I^2^* = 42%; *n* = 3 studies, *n* = 120; GRADE level: moderate). One study per intervention provided preliminary evidence for the positive effects of glutamine supplementation, an Omega-3 PUFA-enriched drink, and education for symptom self-management via a mobile phone game on CIPN symptoms and related outcomes (GRADE: very low). No serious adverse events were reported. The strongest evidence with the most certainty was found for physical exercise as a safe and viable adjuvant to chemotherapy treatment for the prevention and management of CIPN and related physical function in people with advanced cancer. However, the confidence in the evidence to inform conclusions was mostly very low to moderate. Future well-powered and appropriately designed interventions for clinical trials using validated outcome measures and clearly defined populations and strategies are warranted.

## 1. Introduction

Chemotherapy is a commonly used treatment in the advanced stages of cancer as a cancer control strategy, where improvements to symptom management, quality of life, and survival are prioritized [1,2]. However, chemotherapy is often associated with debilitating side effects such as chemotherapy-induced peripheral neuropathy (CIPN), which can lead to suboptimal treatment regimens due to dose reductions or early cessation if the side effects are severe [3]. CIPN, in particular, is highly prevalent and clinically problematic, occurring in up to 60–80% of people receiving chemotherapy [3] as a consequence of neurotoxic damage to the structure and function of peripheral sensory, motor, and autonomic nerves causing peripheral neuropathic symptoms [4]. Greater risk and severity of CIPN is associated with higher doses and longer exposures to neurotoxic chemotherapies, which are characteristic of advanced solid and hematological cancer treatment regimens [3,5].

Sensory CIPN symptoms in the hands and feet are the most common and include pins and needles, pain, hypersensitivity, numbness, itchiness, and hot or cold sensations [6]. Motor CIPN symptoms comprise muscle weakness, wasting, or cramps, as well as impaired motor skills and reflexes, and autonomic CIPN symptoms include gastroparesis as well as cardiac, urogenital, and sexual dysfunction [6]. The severity of CIPN symptoms usually declines after chemotherapy cessation but can also be long-lasting; the pooled prevalence of CIPN symptoms following oxaliplatin treatment for colorectal cancer at 6, 12, 24 and 36 months after chemotherapy completion has recently been reported as 60%, 45%, 30%, and 25%, respectively [7]. Furthermore, people with advanced cancer often have continuous chemotherapy cycles where acute or chronic CIPN is experienced, which presents a unique challenge for this population. Symptoms of CIPN can negatively affect sleep, mood, mobility, activities of daily living, and lead to distress, anxiety, depression, financial toxicity, difficulty feeding, constipation and diarrhea, which further compromise cancer treatment outcomes and quality of life [7,8,9,10,11].

Despite the high prevalence and significant patient and healthcare burden of CIPN, treatment options are limited [3,10,11]. In the most recent 2020 clinical practice guidelines for CIPN developed by the American Society of Clinical Oncology (ASCO) [12] and the European Society for Medical Oncology (ESMO) [13], no recommendations were made for preventing CIPN, and the use of duloxetine was the only treatment described as having some evidence to support its use for reducing neuropathic pain. Despite limited supporting evidence, other pharmacological treatments used in practice include anticonvulsants (e.g., gabapentin/pregabalin), tricyclic antidepressants (e.g., amitriptyline), opioids (e.g., oxycodone), as well as topical lidocaine [4,13,14,15]. Pharmacological interventions target neuropathic pain, which is just one of a cluster of symptoms associated with CIPN [6]. In addition to lack of efficacy and narrow scope of use, pharmaceutical therapies used for CIPN are associated with unfavorable side effects, such as nausea, dizziness, and drowsiness, and include problematic outcomes of polypharmacy interactions with other medications commonly prescribed in advanced cancer due to multimorbidity [4,14]. These toxicities lead to prolonged infusion times, chemotherapy dose reductions, and treatment cessation, which has negative impacts on treatment efficacy and reduces the quality of life [12]. Unsurprisingly, non-pharmacological self-management interventions are of growing interest in assisting in the prevention and management of CIPN [14,16].

Non-pharmacological interventions are generally not first-line therapies for CIPN; however, there is emerging evidence to support their early use [13,16]. A recent systematic review of 24 controlled trials determined non-pharmacological interventions to be more effective than pharmacological treatments in managing Paclitaxel-induced peripheral neuropathy symptoms in people diagnosed with any cancer or stage [14]. Specifically, beneficial effects on CIPN symptoms were described for Chinese herbal medicine, acupuncture, exercise, vitamin E and Omega-3 polyunsaturated fatty acid (PUFA) supplementation, massage, and foot baths [16]. ESMO Guidelines [13] recommend physical exercise as the only non-pharmacological therapy with Level 1 quality evidence for effectively treating neuropathic pain. However, insufficient evidence was available to make strong recommendations, with clinical trials and systematic reviews mostly of poor quality and that did not consider advanced cancer [16].

Non-pharmacological interventions for CIPN that can be self-administered are of particular interest, as symptom self-management strategies are often effective in achieving improved wellbeing, quality of life, self-efficacy, a broader reach of health services, and cost savings in people with cancer [17,18,19]. Self-management strategies can be initiated independently from an internal decision (e.g., massage) or with support from a health professional (e.g., an exercise program) but include only those interventions that the patient is responsible for administering (i.e., not acupuncture) [20,21]. To date, no systematic reviews or clinical guidelines have assessed non-pharmacological self-management strategies specifically for advanced cancer patients, where chemotherapy use and CIPN is common, co-morbidities are high, and daily functioning and overall quality of life are often more valued than enhanced treatment outcomes or survival [22].

Therefore, in people with advanced cancer, this systematic review and meta-analysis of randomized controlled trials sought to determine the effect of non-pharmacological self-management interventions on the incidence and severity of CIPN symptoms and CIPN-related outcomes (quality of life, physical function, sleep, fatigue, gastrointestinal symptoms, nutrition status, psychosocial and financial outcomes, and adverse effects) compared to any control condition.

## 2. Materials and Methods

This systematic review and meta-analysis is registered with the International Prospective Register of Systematic Reviews (PROSPERO ID: 308341) and reported according to the Preferred Reporting Items for Systematic Reviews and Meta-analysis (PRISMA) statement [23].

MEDLINE (Ovid), Embase, Web of Science, CINAHL (EBSCOhost), and Cochrane CENTRAL databases were searched from inception to 7 February 2022 using keywords and controlled vocabulary terms (Supplementary Table S1) based on the following: (“non-pharmacological intervention” AND “chemotherapy-induced peripheral neuropathy” AND (“advanced cancer” OR “metastatic cancer”) AND “randomi?ed controlled trial”). To identify relevant articles not found in the other databases, the first 200 records as sorted by relevance ranking were taken from Google Scholar on 7 February 2022 [24]. A snowballing technique was also used, whereby Google Scholar, database search updates, and reference lists of included studies and relevant literature were assessed to find additional studies not located in the original search strategy up until 5 April 2022.

Automated de-duplication of articles and manual text-mining [25] was conducted by one investigator (MC) using Endnote software (EndNote 20, Clarivate, Version 20.2, London, UK, 2021). During text-mining, irrelevant terms were searched in titles and abstracts to identify studies for exclusion such as ‘systematic review’, ‘protocol’, ‘mice’, ‘conference’, and ‘medication’ [25]. Screening of remaining titles, abstracts and full texts was then completed by two investigators independently (MC and (PMY or AS or OAA)) using Covidence software (Covidence Systematic Review Software, Veritas Health Innovation, Version, Melbourne, Australia, 2021). Screening conflicts were resolved by discussion among the investigators. Studies examining the effect of any non-pharmacological self-management intervention on incidence or severity of CIPN symptoms in people with advanced cancer were included. Advanced cancer was defined as any hematological cancer or any solid tumor with locally or systemically advanced disease (The Union for International Cancer Control Tumor, Nodes, and Metastases Classification of Malignant Tumors (UICC TNM) Stage of III-IV or equivalent) [26]. Self-management strategies were defined as interventions that were administered by the person with cancer or their caregivers, including those initiated with support from a health professional as well as initiated independently [20,21]. Eligibility criteria is presented in Table 1. The primary outcomes were the incidence and severity of CIPN symptoms as measured by any tool at any timepoint. Secondary outcomes were chosen based on existing literature on the factors related to CIPN, including quality of life, physical function, sleep, fatigue, gastrointestinal symptoms, nutrition status, psychosocial and financial outcomes, and adverse effects measured using any tool at any timepoint.

Data pertaining to study, participant, and intervention characteristics, as well as outcomes of interest, were extracted independently by one investigator (MC) and checked for accuracy by a second investigator (OAA). Where outcome data were missing or incompletely reported, investigators contacted the authors via email. Individual study quality assessment was conducted independently by two investigators (MC and OAA) using the Revised Cochrane Risk of Bias Tool for Randomized Trials (RoB2) [27]. The RoB2 comprises five domains with an overall risk of bias judgment calculated as low risk of bias, some concerns, or high risk of bias [27]. Disagreements in data extraction and quality assessment were managed by discussion among investigators.

Certainty in the body of evidence was determined using the Grading of Recommendations, Assessment, Development and Evaluation (GRADE) approach [28] via GRADEpro GDT software (GRADEpro Guideline Development Tool, McMaster University and Evidence Prime, 2021). Four levels of certainty for the estimated effect of each outcome were possible: very low (very little confidence in estimated effect), low (limited confidence), moderate (moderately confident), and high (very confident) [28].

Where two or more studies reported the same intervention and sufficient incidence or mean and variance data for the same outcome, data were pooled by meta-analysis using Review Manager (RevMan) software [RevMan Software, The Cochrane Collaboration, Version 5.4.1, The Cochrane Collaboration, Oxford, UK, 2020]). Using the standard random effects method, categorical outcomes were reported as odds ratios (OR) using the Mantel–Haenszel test, and continuous variables were pooled using the inverse variance test and reported as mean differences (MD), where the same tool and scale were used, or standardized mean differences (SMD), where different measurement tools or scales for the same outcome were used. Interpretation of effects sizes for SMDs was: 0.2 represents a small effect, 0.5 a moderate effect, and 0.8 a large effect [29]. Heterogeneity was evaluated with the I^2^ statistic, whereby >50% represented substantial heterogeneity [30]. The cut-off for statistical significance was considered at a *p*-value of <0.05. Results were synthesized in tabular and narrative format.

## 3. Results

### 3.1. Search Results and Study Quality

**Figure 1:**
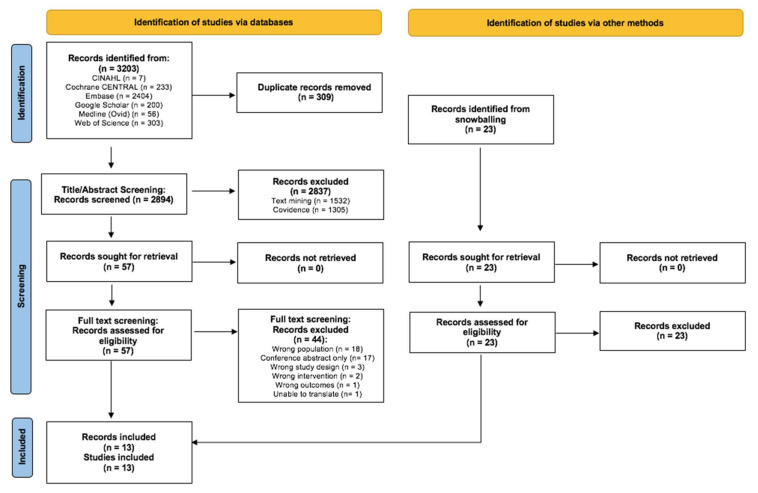
Preferred Reporting Items for Systematic Reviews and Meta-analyses (PRISMA) diagram for selecting studies that examined the effect of non-pharmacological self-management interventions on chemotherapy-induced peripheral neuropathy symptoms and related outcomes in people with advanced cancer.

**Figure 2:**
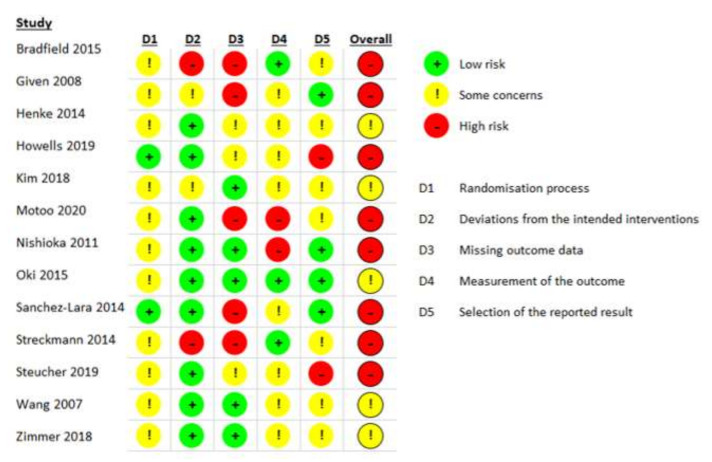
The Revised Cochrane Risk of Bias Tool for Randomized Trials (RoB2) assessment of studies that examined the effect of non-pharmacological self-management interventions on chemotherapy-induced peripheral neuropathy symptoms and related outcomes in people with advanced cancer [31,32,33,34,35,36,37,38,39,40,41,42,43].

Thirteen studies [31,32,33,34,35,36,37,38,39,40,41,42,43] published between 2014 and 2020 were included (Figure 1). As indicated in Figure 2, 62% of studies had high risk of bias [32,33,35,36,37,39,40,42] and the remaining 38% had unclear risk of bias [31,34,38,41,43]. One main reason for bias was the lack of outcome assessor blinding and the use of subjective outcome measurement tools that could have influenced outcome assessment due to the knowledge of the intervention received. However, this is an acknowledged limitation in most exercise, diet, and lifestyle research [44]. Other common sources of potential bias were the lack of information regarding allocation concealment procedures, failure to consider missing data with methods such as intention-to-treat and imputation, selective outcome reporting, and inadequate reporting of whether the study was conducted in accordance with a retrospective protocol. Publication bias was unable to be assessed due to the small number of studies in each meta-analysis. GRADE level of evidence for all outcomes was very low to low.

### 3.2. Study Samples

Characteristics of the 13 studies are summarized in Table 2 and detailed in Supplementary Table S2. Study samples ranged from *n* = 27 [36] to *n* = 200 [35], representing a total sample of N = 1012 participants [31,32,33,34,35,36,37,38,39,40,41,42,43]. One study comprised pediatrics [35], while the remaining studies focused on adults only [31,32,33,34,36,37,38,39,40,41,42,43]. The majority of studies (85%) included patients with advanced solid tumors [31,32,34,36,37,38,39,40,41,42,43], most of which (46%) had colorectal cancer [34,36,38,39,40,41]. Most studies (92%) included patients undergoing active chemotherapy [31,32,33,35,36,37,38,39,40,41,42,43], and the remaining study (8%) [34] included those undergoing or who completed chemotherapy. Of the 12 studies (92%) that reported chemotherapy type, all used neurotoxic agents [31,33,34,35,36,37,38,39,40,41,42,43] and all of the seven studies (54%) that reported CIPN history only included participants with no existing CIPN [32,35,36,38,39,40,41].

### 3.3. Intervention Characteristics

Four studies (31%) examined physical exercise interventions (strength, endurance, and sensorimotor training (*n* = 2) [33,34], strength and endurance training (*n* = 1) [31], and walking (*n* = 1) [32]); four studies (31%) assessed nutrition supplements (glutamatic acid (*n* = 1) [35], glutamine (*n* = 1) [38], curcumin (*n* = 1) [36], and Omega-3 PUFA-enriched nutrition drink (*n* = 1) [37]); three studies (23%) explored orally consumed Japanese herbal medicine (Goshajinkigan (*n* = 2) [40,41] and ninjin’yoeito (*n* = 1) [39]); and two studies (15%) assessed technology-facilitated education for symptom self-management (education for self-management via automated telephone voice technology incorporating symptom monitoring (*n* = 1) [42] and education for symptom self-management via a mobile phone game (*n* = 1) [43]). Comparator groups included standard care (55%) [31,32,33,36,38,39,40], placebo (15%) [35,41], written information (15%) [34,43], or an active control (15%) [37,42].

### 3.4. Effect on CIPN Symptoms and Related Outcomes

Six studies (46%) [31,32,34,39,40,41] were analyzed in 18 meta-analyses. Table 3 summarizes meta-analysis results, and forest plots for non-significant findings are presented in Supplementary Figures S1–S3. Justifications for GRADE ratings are given in Supplementary Table S3.

#### 3.4.1. Physical Exercise

**Figure 3:**
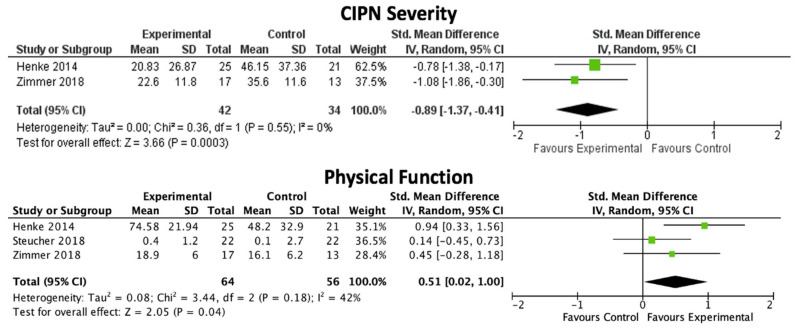
Severity of CIPN was significantly less and physical function was significantly higher with physical exercise compared to standard care or written exercise guidelines in people with advanced lung or gastrointestinal cancer (GRADE level: moderate) [31,32,34].

Incidence of CIPN was significantly lower with a strength, endurance, and sensorimotor training program in comparison to standard care (*n* = 1 study [33]; *n* = 61 participants with lymphoma; GRADE level: very low). Severity of CIPN was significantly less with strength and endurance training with or without sensorimotor training when compared to standard care or written exercise guidelines via meta-analysis (SMD: −0.89, 95% CI: −1.37 to −0.41; *p* = 0.0003; *I^2^* = 0%; *n* = 2 studies [31,34], *n* = 76 participants with lung or colorectal cancer; GRADE level: moderate; Figure 3).

Physical function was significantly higher with exercise (strength and endurance training with or without sensorimotor training, or walking) when compared to standard care or written exercise guidelines via meta-analysis (SMD: 0.51, 95% CI: 0.02 to 1.00; *p* = 0.04; *I^2^* = 42%; *n* = 3 studies [31,32,34], *n* = 120 participants with lung or gastrointestinal cancer; GRADE level: moderate; Figure 3). Quality of life [31] and emotional wellbeing [33] were significantly higher with exercise in one of three studies (33%), but no significant effect was found with meta-analysis (*n* = 76 participants with lung or colorectal cancer; GRADE level: very low). Significant improvements in pain [31], sleep [33], fatigue [33], diarrhea [33], and financial problems [33] were found with exercise in one of two studies (50%), but data were unable to be pooled by meta-analysis (*n* = 107 participants with lymphoma or lung cancer; GRADE level: very low). One study found improvements in nutrition status and lean body mass with walking (*n* = 44 participants with gastrointestinal cancer; GRADE level: very low). No serious adverse events were reported with exercise interventions [32,33,34].

#### 3.4.2. Nutrition Supplements

Incidence of CIPN was significantly lower with glutamine supplementation compared to standard care (*n* = 1 study [38]; *n* = 86 participants with colorectal cancer; GRADE level: very low), whereas participants prescribed curcumin had a significantly higher incidence of CIPN in comparison to standard care (*n* = 1 study [36]; *n* = 27 participants with colorectal cancer; GRADE level: very low). CIPN severity was significantly lower with consumption of an Omega-3 PUFA-enriched nutrition drink on an isocaloric diet compared to an isocaloric diet alone (*n* = 1 study [37]; *n* = 112 participants with lung cancer; GRADE level: very low).

Quality of life and incidence of chemotherapy dose reduction was significantly improved with glutamine consumption compared to standard care (*n* = 1 study [38]; *n* = 86 participants with colorectal cancer; GRADE level: very low). Fatigue severity, appetite loss, and body weight maintenance were significantly improved with the consumption of an Omega-3 PUFA-enriched drink on an isocaloric diet compared to an isocaloric diet alone (*n* = 1 study [37]; *n* = 112 participants with lung cancer; GRADE level: very low). Curcumin supplementation was associated with a significantly higher cancer treatment response rate and length of survival but also led to a significantly increased incidence of diarrhea (*n* = 1 study [36]; *n* = 27 participants with colorectal cancer; GRADE level: very low). No serious adverse events were reported with nutrition supplements [36,37,38].

#### 3.4.3. Japanese Herbal Medicine

The incidence of CIPN was significantly reduced with the consumption of Japanese herbal medicine in two studies [39,40], while the remaining study [41] found a significantly increased incidence with the intervention. However, when meta-analyzed, Japanese herbal medicine had no significant association with the likelihood of CIPN of Grade 1, 2, 3 or ≥2 (*n* = 3 studies [39,40,41]; *n* = 283 participants with colorectal cancer; GRADE level: very low). Sensitivity analysis according to the type of herbal supplement (Goshajinkigan vs. ninjin’yoeito) did not result in significant findings.

Relative dose intensity of oxaliplatin was significantly higher with consumption of Japanese herbal medicine in two [39,41] of three studies [39,40,41]; however, there was no significant effect with meta-analysis (*n* = 2 studies [39,41]; *n* = 238 participants with colorectal cancer; GRADE level: very low). No serious adverse events were reported with the consumption of Japanese herbal medicine [39,40,41].

#### 3.4.4. Technology-Facilitated Education for Symptom Self-Management

The incidence of CIPN was significantly lower in participants who received education for symptom self-management via a mobile phone game in comparison to a symptom management booklet (*n* = 1 study [43]; *n* = 76 participants with breast cancer; GRADE level: very low). Time to CIPN symptom response was lower in participants who received education via automated telephone voice technology incorporating symptom monitoring compared to cognitive-behavioral nurse-administered symptom management via telephone (35 vs. >55 days); however, statistical significance was not tested (*n* = 1 study [42]; *n* = 47 participants with breast cancer; GRADE level: very low).

Quality of life and medication compliance was significantly higher in participants who received education for symptom self-management via a mobile phone game in comparison to a symptom management booklet; however, results for physical function, nausea, and psychological health favored the control (*n* = 1 study [43]; *n* = 76 participants with breast cancer; GRADE level: very low). No serious adverse events were reported [43].

## 4. Discussion

This systematic review is the first to synthesize evidence on the non-pharmacological self-management interventions for CIPN and related outcomes in people with advanced cancer. The strongest evidence was found for physical exercise with medium to large beneficial effects on CIPN severity and physical function in adults, with moderate certainty in the estimated effect size [31,34]. Some evidence with very low certainty in the effect, and supported by one study only, was found in adults for the positive effects of glutamine supplementation on CIPN incidence, quality of life, and incidence of chemotherapy dose reduction [38]; an Omega-3 PUFA-enriched nutrition drink for CIPN severity, fatigue, appetite loss, and body weight maintenance [37]; and a symptom self-management mobile phone game for CIPN incidence, quality of life, and medication compliance [43]. Findings are inconclusive as to whether CIPN symptoms are improved by glutamatic acid supplementation in children [35] and curcumin supplementation [36], Japanese herbal medicine consumption [39,40,41], and an automated telephone symptom management system [42] in adults. There were no serious adverse events reported with any non-pharmacological self-management interventions [31,32,33,34,35,36,37,38,39,40,41,42,43].

Most evidentiary support exists for physical exercise for CIPN in people with advanced cancer, which has also been reported in the general cancer population. This is consistent with the findings from a recent systematic review [45] of 16 physical exercise interventions for CIPN in people at any stage of cancer, where exercise also improved physical function. The present review found additional benefits of exercise on CIPN symptom severity, which is unique to our review and perhaps unique to people with advanced cancer, and was not a finding in the general cancer population [45]. These beneficial effects of exercise on CIPN are supported by mechanistic studies. For example, exercise counteracts the deleterious effects of chemotherapy on the nervous system central to CIPN by reducing inflammation, suppressing pain pathways, and enhancing neuroprotective factors involved in the development, survival, and function of neurons [46,47]. In addition, exercise has broader health benefits for people with advanced cancer, such as improved quality of life, fatigue, body composition, psychosocial function, and sleep quality, and it appears safe when implemented on advice from a health professional [48]. Although strong recommendations cannot be made with the available evidence for the use of exercise for CIPN symptoms and related outcomes, it appears to be a safe, cost-effective, and viable adjuvant to chemotherapy to promote general health and wellbeing as well as CIPN prevention and management. Future well-powered randomized controlled trials are needed to confirm the efficacy and determine optimal exercise regimens, including exercise type and frequency, which is largely heterogeneous in the existing literature.

An emerging area of research for the prevention and management of CIPN in advanced cancer is the use of nutritional supplements. Consistent with the findings of this review, a recent meta-analysis in the general cancer population found that participants consuming Omega-3 PUFA supplements were 80% less likely to experience CIPN [49]. However, the findings of our current review were based on a study supplementing Omega-3 PUFA in an oral nutrition supplement drink [37]. Therefore, it cannot be confirmed whether the beneficial effects on CIPN resulted from Omega-3 PUFA or one or a combination of other nutrients with known benefits on nerve functioning and suggested modest benefits on CIPN in other cancer populations, such as amino acids, B vitamins, vitamin E, and magnesium [16,50,51,52]. Our current review did find additional potential benefits of the amino acid glutamine for CIPN in advanced cancer populations [38]. Elsewhere, a review of five studies found oral glutamine to benefit neuropathic pain in participants with cancer of any stage; however, the clinical efficacy of glutamine for CIPN was judged to be unable to justify the additional daily cost [53]. Thus, future research on dietary interventions should consider cost-effectiveness in conjunction with the efficacy of isolated compounds and prioritize assessment of essential nutrients rather than non-nutrients such as curcumin or Japanese herbal medicine, for which there is less convincing evidence. No clinical recommendations can yet be made for nutrition supplements for CIPN in advanced cancer patients. However, clinical practice should focus on correcting nutritional deficiencies prior to, during, and after chemotherapy, especially for the aforementioned nutrients with suggested involvement in CIPN or biochemical parameters that have been found to be deficient in those with CIPN, such as vitamin D, hemoglobin, and albumin [54,55].

Our review also suggests that technology could assist in the education for self-management of CIPN. In the general cancer population, healthcare interventions delivered via a mobile device (mHealth) have been associated with improved pain, fatigue, psychological distress, and sleep outcomes [56]. mHealth interventions have also shown potential to reach a large population due to ease of access, resulting in economic benefits to patients and healthcare systems [56]. In people with advanced cancer, improvements in cancer-related pain were observed after mHealth enabled psychoeducation that would otherwise only be accessible when implemented by a specialist [57]. Technology aside, the research emphasizes the requirement for self-management strategies in people with advanced cancer to be individualized and multi-faceted to consider older age, depression, impaired physical functioning, and low literacy, which may lead to greater difficulty in symptom self-management [17]. In addition, self-management strategies should be embedded within the person’s support network and include strong partnerships with healthcare professionals, caregivers, and relatives [17]. Future research is warranted to determine the key elements of self-management strategies and the potential role of technology to facilitate implementation specifically for CIPN in people with advanced cancer.

### Limitations and Future Research

Conclusions of this review have been drawn from a limited number of heterogeneous studies, evident by most outcomes having a GRADE rating of very low. Reasons for downgrading the level of evidence included substantial heterogeneity, small sample size, inadequate blinding, unclear random allocation concealment, lack of intention-to-treat analysis, and selective outcome reporting. Sources of heterogeneity were varying chemotherapy regimens, cancer types, and interventions, which were often reported with minimal detail. Furthermore, CIPN assessment methods were mostly subjective, not always validated, and differed greatly between the studies, which is problematic as a wide variation in CIPN outcomes has been noted with different measurement tools [58]. This suggests that the current tools measure different aspects of CIPN and thus might need to be implemented in combination to gather an accurate evaluation of CIPN. In addition, this systematic review was stringently conducted and reported according to best-practice guidelines [23,29] but only included studies in English and, therefore, may not have captured valuable research from some countries.

Future studies are needed to enhance the confidence in findings by increasing the body of evidence as well as the quality of the conduct of research. Randomized controlled trials should be well-designed with prescriptive interventions that seek to establish cause-effect and dose–response relationships. Randomized controlled trials should also be well-powered and use validated outcome measures and blinding where possible. Data should be reported in full and include adverse events, secondary outcomes related to CIPN, and compliance with self-management interventions. In people with advanced cancer, studies should prioritize exercise interventions and consider nutritional interventions and interactive technology-facilitated education for self-management support strategies administered alone or in combination. Additional suggested areas of research include those which have shown benefit in the general cancer population but are yet to be tested in advanced cancer. This includes low-cost and easily accessible interventions of massage, heat therapy, and meditation [20,59]. Future studies should also explore how non-pharmacological self-management interventions may best be implemented in conventional cancer care.

## 5. Conclusions

The strongest evidence with the most certainty was found for physical exercise as a safe and viable adjuvant to chemotherapy treatment for the prevention and management of CIPN and related physical function in people with advanced cancer. Nutrition supplements of glutamine and an Omega-3 PUFA-enriched drink showed some benefit, and the use of interactive technology may facilitate education for self-management of CIPN; however, certainty of these effects was very low. The confidence in the evidence to inform conclusions was mostly very low to moderate, which warrants future clinical trials. Trials should be rigorously designed and reported to include adequate sample size, clearly defined populations and interventions, and use valid outcome measures.

## Figures and Tables

**Table 1:** Eligibility criteria for studies assessing the effect of non-pharmacological self-management interventions on chemotherapy-induced peripheral neuropathy symptoms and related outcomes in people with advanced cancer.

	Inclusion Criteria	Exclusion Criteria
Population	Advanced cancer: any solid tumor with locally or systemically advanced disease stage (UICC TNM stage III–IV) [26] or any hematological cancer ^1^ Undergoing or have undergone chemotherapy Any age (i.e., adults and children)	Studies including people at any cancer stage where results have not been reported separately for those with advanced cancer
Intervention	Any non-pharmacological self-management intervention administered by the person with cancer or their caregiver (e.g., exercise, oral nutrition supplements, massage, thermal therapies, meditation), including those initiated with support from a health professional	Pharmacological interventions (e.g., prescribed and non-prescribed medications) Eligible non-pharmacological interventions administered in conjunction with pharmacological interventions for CIPN (e.g., duloxetine, gabapentin, pregabalin, carbamazepine, amitriptyline) Non-pharmacological interventions not administered by the person with cancer (e.g., acupuncture, electrical nerve stimulation, scrambler therapy, moxibustion, reflexology, intravenous vitamins)
Comparator	Any control (e.g., placebo, usual care)	
Outcomes	Incidence or severity of CIPN symptoms as measured by any tool	
Study design	Randomized controlled parallel trials Published in peer-reviewed journals	Conference abstracts only Non-randomized controlled trials Narrative/systematic reviews, qualitative studies, protocols, case studies, observational studies
Language	English or able to be translated into English	

UICC TNM: The Union for International Cancer Control Tumor, Nodes, and Metastases Classification of Malignant Tumors. ^1^ Studies that referred to ‘advanced cancer’ but do not specify the stage and/or type of cancer were eligible for inclusion.

**Table 2:** Summary of characteristics and findings of studies that examined the effect of non-pharmacological self-management interventions on chemotherapy-induced peripheral neuropathy symptoms and related outcomes in people with advanced cancer.

Study and Population Characteristics				Intervention Characteristics		Findings														
						CIPN			CIPN-Related Outcomes											
Citation and Country	Population	Cancer	CTX	Intervention	Control	Tool and Time Point	Incidence	Severity	Quality of Life	Physical function	Pain	Sleep	Fatigue	GI symptoms	Nutrition status	Psychological	Social	Treatment	Financial	Adverse events
Physical exercise																				
Henke 2014 [31]; Germany	N: 46 Age (yrs): NR Males: NR	Type: lung Stage: ≥3 Existing CIPN: NR	Type: Platinum-based Frequency: NR Duration: NR Status: ongoing	Strategy: Strength and endurance training (*n* = 25) Regimen: 8 min endurance or 3 sets of 4 strength exercises daily Duration: 3 CTX cycles, from C1D1	Standard care (*n* = 21)	EORTC QLQ-LC13; pre and post (C3)		+	+	+	+	o	o	o		o	o		o	
Stuecher 2019 [32]; Germany	N: 44 Age (yrs): 67 ± 8 Males: 67%	Type: gastrointestinal Stage: ≥3 Existing CIPN: no	Type: NR Frequency: NR Duration: NR Status: ongoing	Strategy: Walking (*n* = 22) Regimen: 150 min per wk Duration: 12 wks, from C1D1	Standard care (*n* = 22)	Tuning fork test; pre and post (6 and 12 wks)		o		+					+					o
Streckmann 2014 [33]; Germany	N: 61 Age (yrs): 46 (19–73) Males: 77%	Type: lymphoma Stage: progressive Existing CIPN: NR	Type: mixed Frequency: NR Duration: NR Status: ongoing	Strategy: Strength, endurance, and sensorimotor training (*n* = 30) Regimen: 1 h session twice per wk Duration: 36 wks	Standard care (*n* = 31)	Tuning fork test; pre and post (12, 24 and 36 wks)	+		o	+	o	+	+	+		+			+	o
Zimmer 2018 [34]; Germany	N: 30 Age (yrs): 50–81 Males: 70%	Type: colorectal Stage: 4 Existing CIPN: NR	Type: mixed Frequency: NR Duration: 2–3 cycles Status: ongoing and ceased	Strategy: Strength, endurance, and sensorimotor training (*n* = 17) Regimen: 1 hr session twice per wk Duration: 8 wks	Written exercise guidelines (*n* = 13)	FACT/GOG-NTX; pre and post (8 and 12 wks)		+	o	+						o	o			o
Nutrition supplements																				
Bradfield, 2015 [35]; USA	N: 200 Age (yrs): 9 ± 5 Males: 62%	Type: lymphoma Stage: NR Existing CIPN: no	Type: Vincristine Frequency: weekly Duration: ≥4 wks Status: ongoing	Strategy: L-glutamic acid in capsule form, taken orally (*n* = 101) Regimen: 3 times daily, total 0.75–1.5 g per day Duration: 5 wks	Placebo (*n* = 99)	mBPSPN; pre and post (5 wks)	o													
Howells, 2019 [36]; UK	N: 27 Age (yrs): 68 (53–78) Males: NR	Type: colorectal Stage: metastatic Existing CIPN: no	Type: 5FU and oxaliplatin Frequency: fortnightly Duration: ≤12 cycles Status: ongoing	Strategy: Curcumin powder in capsule form, taken orally (*n* = 18) Regimen: 4 times daily, total 2 g per day Duration: duration of CTX (from 7 days before C1D1)	Standard care (*n* = 9)	EORTC-QLQ-C30 and NCI-CTAE; pre and post	-	o	o	o	o	o	o	-	o			+		o
Sanchez-Lara, 2014 [37]; Mexico	N: 112 Age (yrs): 18–80 Males: 47%	Type: NSCL Stage: ≥3 b Existing CIPN: NR	Type: paclitaxel and cisplatin/carboplatin Frequency: every wks Duration: 2–6 cycles Status: ongoing	Strategy: omega 3 (EPA)-enriched oral nutrition supplement + isocaloric diet (*n* = 54) Regimen: 2 237 mL drinks per day (provides 2.2 g EPA) Duration: 2 CTX cycles, from C1D1	Isocaloric diet (*n* = 58)	EORTC-QLQ-C30 and -LC13; pre and post (C1 and C2)		+	o	o			+	+	+			o		o
Wang, 2007 [38]; Taiwan	N: 86 Age (yrs): 60% ≥50 Males: 65%	Type: colorectal Stage: metastatic Existing CIPN: no	Type: 5FU and oxaliplatin Frequency: every 4 wks Duration: NR Status: ongoing	Strategy: Levo-Glutamine, taken orally (*n* = 42) Regimen: twice daily, total 30 g per day for 7 days every 2 wks Duration: 6 cycles, from C1D1	Standard care (*n* = 44)	NCI-CTCAE and Electro-physiological exam; pre and post (C2, C4 and C6)	+		+									+		o
Japanese herbal medicine																				
Motoo 2020 [39]; Japan	N: 52 Age (yrs): 35–79 Males: 60%	Type: colorectal Stage: 3 Existing CIPN: no	Type: capecitabine and oxaliplatin Frequency: every 3 wks Duration: 8 cycles Status: ongoing	Strategy: ninjin’yoeito powder ^1^, taken orally (*n* = 26) Regimen: 2–3 times daily, total 9 g per day Duration: 8 cycles, from C1D1	Standard care (*n* = 26)	NCI-CTCAE; pre and post (C1–C8)	+					o	o	o				+		o
Niskioka, 2011 [40]; Japan	N: 45 Age (yrs): 48–80 Males: 49%	Type: colorectal Stage: metastatic Existing CIPN: no	Type: 5FU and oxaliplatin Frequency: every 2 wks Duration: 4–32 cycles Status: ongoing	Strategy: Goshajinkigan ^2^, taken orally (*n* = 22) Regimen: 2–3 times daily, total 7.5 g per day Duration: entire CTX course (4–32 cycles), from C1D1	Standard care (*n* = 23)	DEB-NTC; pre and post (at each CTX cycle)	+							o				o		o
Oki, 2015 [41]; Japan	N: 186 Age (yrs): 61 ± 11 Males: 55%	Type: colorectal Stage: 3 Existing CIPN: no	Type: 5FU and oxaliplatin Frequency: every 2 wks Duration: 12 cycles Status: ongoing	Strategy: Goshajinkigan ^2^, taken orally (*n* = 93) Regimen: daily with meals, total 7.5 g per day Duration: entire CTX course (12 cycles), from C1D1	Placebo (*n* = 93)	NCI-CTCAE and DEB-NTC; pre and post (at each CTX cycle)	-						o	o				+		o
Technology-facilitated education for symptom self-management																				
Given, 2008 [42]; USA	N: 47 Age (yrs): ≥21 Males: 0%	Type: breast Stage: metastatic Existing CIPN: NR	Type: mixed Frequency: NR Duration: NR Status: ongoing	Strategy: Education for symptom self-management via automated telephone voice technology incorporating symptom monitoring (*n* = 24) Regimen: weekly phone calls for 4 wks, then at wk 6 and wk 8 Duration: 8 wks	Cognitive behavioral nurse-administered telephone symptom management (*n* = 23)	11-point Likert scale; pre and post (10 and 16 wks)	?			?	?	?	?	?		?				
Kim, 2018 [43]; Korea	N: 76 Age (yrs): 51 ± 7 Males: 0%	Type: breast Stage: 4 Existing CIPN: NR	Type: mixed Frequency: NR Duration: NR Status: ongoing	Strategy: Education for symptom self-management via a mobile phone game (*n* = 36) Regimen: >30 min per day, 3 times per wk Duration: 3 wks	Symptom management booklet (*n* = 40)	NCI-CTCAE; pre and post (3 wks)	+		+	-				-		-	o	+		o

^1^ Contains 12 crude Japanese herbs: Rehmannia root, Angelica root, Atractylodes rhizome, Poria Sclerotium, Ginseng, Cinnamon bark, Polygala root, Peony root, Citrus Unshiu peel, Atsragalus root, Glycyrrhiza, Schisandra fruit. ^2^ Contains 10 crude Japanese herbs: Rehmannia root, Achyranthes root, Cornus fruit, Dioscorea rhizome, Plantago seed, Alisma Rhizome, Poria Sclerotium, Moutan bark, Cinnamon bark, and aconite root. 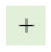. Statistically significant positive effect favoring intervention. 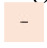. Statistically significant negative effect favoring control.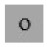. No statistically significant effect. 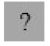. Statistical significance not tested. 5FU: Fluorouracil; C: chemotherapy cycle; CIPN: chemotherapy-induced peripheral neuropathy; CTX: chemotherapy; D: day; DEB-NTC: Neurotoxicity Criteria of Debiopharm; EORTC QLQ-C30: European Organisation for Research and Treatment of Cancer Quality of Life Questionnaire; EORTC QLQ-LC13: European Organization for Research and Treatment of Cancer Quality of Life Questionnaire Lung Cancer 13; EPA: eicosapentaenoic acid; FACT/GOG-NTX: Functional Assessment of Cancer Therapy Gynecologic Oncology Group Neurotoxicity; GI: Gastrointestinal; hr: hour; min: minutes; mBPSPN: Modified Balis Pediatric Scale of Peripheral Neuropathies; NCI-CTCAE: National Cancer Institute Common Terminology Criteria for Adverse Events; NSCL: non-small cell lung cancer; NR: not reported; UK: United Kingdom; USA: United States of America; wk: week; yrs: years.

**Table 3:** Results from meta-analyses that were conducted to evaluate the effect of non-pharmacological self-management interventions on chemotherapy-induced peripheral neuropathy symptoms and related outcomes in people with advanced cancer.

Outcome	Pooled Estimate	Significance of Pooled Estimate	Heterogeneity	Number of Studies (Citations)	Sample Size	GRADE Level of Evidence
Physical exercise						
CIPN severity	SMD: −0.89, 95% CI: −1.37, −0.41	*p* = 0.0003	0%	2 [31,34]	76	Moderate
Quality of life	SMD: 0.47, 95% CI: 0.01, 0.93	*p* = 0.05	0%	2 [31,34]	76	Very low
Physical function	SMD: 0.51, 95% CI: 0.02, 1.00	*p* = 0.04	42%	3 [31,32,34]	120	Moderate
Endurance	SMD: 1.11, 95% CI: −0.65, 2.87	*p* = 0.22	93%	2 [31,34]	76	Very low
Emotional wellbeing	SMD: 0.21, 95% CI: −0.42, 0.83	*p* = 0.52	45%	2 [31,34]	76	Very low
Social wellbeing	SMD: −0.02, 95% CI: −0.53, 0.50	*p* = 0.95	21%	2 [31,34]	76	Very low
Japanese herbal medicine						
CIPN incidence - Grade 1	OR: 1.98, 95% CI: 0.08, 48.14	*p* = 0.68	92%	2 [39,41]	226	Very low
CIPN incidence - Grade 2	OR: 0.64, 95% CI: 0.06, 6.71	*p* = 0.71	85%	2 [39,41]	226	Very low
CIPN incidence - Grade 3	OR: 0.37, 95% CI: 0.05, 2.52	*p* = 0.31	80%	3 [39,40,41]	271	Very low
CIPN incidence - Grade 2 and 3	OR: 0.23, 95% CI: 0.01, 3.89	*p* = 0.31	89%	3 [39,40,41]	271	Very low
Fatigue	OR: 0.40, 95% CI: 0.06, 2.93	*p* = 0.37	70%	2 [39,41]	238	Very low
Nausea	OR: 0.80, 95% CI: 0.28, 2.23	*p* = 0.66	30%	3 [39,40,41]	283	Very low
Vomiting	OR: 0.63, 95% CI: 0.34, 1.16	*p* = 0.13	0%	3 [39,40,41]	283	Very low
Diarrhoea	OR: 1.20, 95% CI: 0.67, 2.17	*p* = 0.54	0%	2 [40,41]	231	Very low
Anorexia	OR: 0.71, 95% CI: 0.39, 1.27	*p* = 0.25	0%	3 [39,40,41]	283	Very low
Relative dose intensity of oxaliplatin	SMD: 1.77, 95% CI: −1.13, 4.68	*p* = 0.23	98%	2 [39,41]	238	Very low
Side effect: Neutropenia	OR: 0.74, 95% CI: 0.41, 1.31	*p* = 0.30	0%	3 [39,40,41]	283	Very low
Side effect: Thrombocytopenia	OR: 1.52, 95% CI: 0.87, 2.67	*p* = 0.14	0%	2 [39,41]	238	Very low

## Supplementary Materials

The following supporting information can be downloaded at: https://www.mdpi.com/article/10.3390/nu14122403/s1, Table S1: Systematic search strategy to identify randomized controlled trials that examined the effect of non-pharmacological interventions on chemotherapy-induced peripheral neuropathy symptoms and related outcomes; Table S2: Characteristics and findings of studies that examined the effect of self-administered non-pharmacological interventions on chemotherapy-induced peripheral neuropathy symptoms and related outcomes; Figure S1: Japanese herbal medicine had no significant association with the likelihood of CIPN of Grade 1, 2, 3 or ≥2 (*n* = 2–3 studies; *n* = 226–271 participants; GRADE level: very low). Sensitivity analysis according to the type of herbal supplement (Goshajinkigan vs. ninjin’yoeito) did not result in significant findings; Figure S2: Physical exercise had no significant effect on quality of life, endurance, and emotional or social wellbeing (*n* = 2 studies; *n* = 76 participants; GRADE level: very low); Figure S3: Japanese herbal medicine had no significant effect on fatigue, nausea, vomiting, diarrhea, anorexia, relative dose reduction in oxaliplatin, neutropenia, nor thrombocytopenia (*n* = 2–3 studies; *n* = 231–283 participants; GRADE level: very low); Table S3: Grading of Recommendations, Assessment, Development and Evaluation (GRADE) for each outcome examining the effect of self-administered non-pharmacological interventions on chemotherapy-induced peripheral neuropathy symptoms and related outcomes.
