# Supplementary material for: Non-Pharmacological Self-Management Strategies for Chemotherapy-Induced Peripheral Neuropathy in People with Advanced Cancer: A Systematic Review and Meta-Analysis
Source: Nutrients. 2022 Jun 9;14(12):2403. doi: 10.3390/nu14122403 (PMC9228711; doi:10.3390/nu14122403)
Supplement: Supplementary file 1 [file nutrients-14-02403-s001.zip › nutrients-1745029-supplementary.pdf]

Non-pharmacological self-management strategies for chemotherapy-induced peripheral neuropathy in people with advanced cancer: A systematic review and meta-analysis

Megan Crichton, Patsy M Yates, Oluwaseyifunmi A Agbejule, Amy Spooner, Raymond J Chan, Nicolas H Hart

Supplementary Material

Supplementary File S1. Systematic search strategy

Supplementary Table S1. Systematic search strategy to identify randomized controlled trials that examined the effect of non-pharmacological interventions on chemotherapy-induced peripheral neuropathy symptoms and related outcomes.

**MEDLINE (via Ovid)**	(neoplasms.sh. or neoplasm*.ti. or oncolog*.ti. or carcinoma*.ti. or tumour*.ti. or tumor*.ti. or cancer*.ti. or malignanc*.ti. or neoplasm*.ab. or oncolog*.ab. or carcinoma*.ab. or tumour*.ab. or tumor*.ab. or cancer*.ab. or malignanc*.ab.)<br>AND<br>(("life style" or exercise or "physical therapy modalities" or "behavior control" or "complementary therapies" or cryotherapy or "nutrition therapy" or balneology or "hyperthermia, induced" or "food and beverages" or "diet, food, and nutrition" or "herbal medicine" or "plant preparations").sh. or nonpharmacologic*.ab. or nonpharmacologic*.ti. or "non pharmacologic*".ab. or "non pharmacologic*".ti. or physical*.ab. or physical*.ti. or exercise*.ab. or exercise*.ti. or yoga.ab. or yoga.ti. or "tai chi".ab. or "tai chi".ti. or imagery.ab. or imagery.ti. or "art therap*".ab. or "art therap*".ti. or "mind body".ab. or "mind body".ti. or "mind and body".ab. or "mind and body".ti. or mindful*.ab. or mindful*.ti. or meditation.ab. or meditation.ti. or behaviour*.ab. or behaviour*.ti. or behavior*.ab. or behavior*.ti. or lifestyle*.ab. or lifestyle*.ti. or massage.ab. or massage.ti. or "foot bath".ab. or "foot bath".ti. or hydrotherapy.ab. or hydrotherapy.ti. or cryotherap*.ab. or cryotherap*.ti. or complementary*.ab. or complementary*.ti. or cam.ab. or cam.ti. or acupressure.ab. or acupressure.ti. or vitamin*.ab. or vitamin*.ti. or mineral*.ab. or mineral*.ti. or herbal*.ab. or herbal*.ti. or supplement*.ab. or supplement*.ti. or natur*.ab. or natur*.ti. or homeopath*.ab. or homeopath*.ti. or nutraceutical*.ab. or nutraceutical*.ti. or "chinese medicine*".ab. or "chinese medicine*".ti. or nutrition*.ab. or nutrition*.ti. or botanical*.ab. or botanical*.ti. or cannab*.ab. or cannab*.ti.)<br>AND<br>("peripheral nervous system diseases".sh. or neuralgia.ab. or paresthesia.ab. or paraesthesia.ab. or hyperalgesia.ab. or cipn.ab. or neuropath*.ab. or polyneuropath*.ab. or neuralgia.ti. or paresthesia.ti. or paraesthesia.ti. or hyperalgesia.ti. or cipn.ti. or neuropath*.ti. or polyneuropath*.ti.)<br>AND<br>(("neoplasm metastasis" or "palliative care" or "terminal care" or "palliative medicine" or "hospice and palliative care nursing" or "terminally ill" or "hematologic neoplasms").sh. or advanced.ab. or advanced.ti. or metasta*.ab. or metasta*.ti. or palliati*.ab. or palliati*.ti. or terminal.ab. or terminal.ti. or "end of life".ab. or "end of life".ti. or incurable.ab. or incurable.ti. or

	hospice.ab. or hospice.ti. or hematolog*.ab. or hematolog*.ti. or haematolog*.ab. or haematolog*.ti.)<br>AND<br>(PT "randomized controlled trial" OR TI randomized OR AB randomized OR TI randomised OR AB randomised OR TI RCT OR AB RCT OR TI controlled OR AB controlled)
**Embase**	(‘Neoplasm’/exp OR neoplasm*:ab,ti OR oncolog*:ab,ti OR carcinoma*:ab,ti OR tumour*:ab,ti OR tumor*:ab,ti OR cancer*:ab,ti OR malignanc*:ab,ti)<br>AND<br>(‘Lifestyle’/exp OR ‘Exercise’/exp OR ‘Behavior Control’/exp OR ‘Alternative medicine’/exp OR ‘Cryotherapy’/exp OR ‘diet Therapy’/exp OR ‘Balneotherapy’/exp OR ‘thermotherapy’/exp OR ‘food’/exp OR ‘nutrition’/exp OR ‘Herbal Medicine’/exp OR ‘plant medicinal product’/exp OR nonpharmacologic*:ab,ti OR “non pharmacologic*”:ab,ti OR physical*:ab,ti OR exercise*:ab,ti OR yoga:ab,ti OR “tai chi”:ab,ti OR imagery:ab,ti OR “art therap*”:ab,ti OR “mind and body”:ab,ti OR “mind body”:ab,ti OR mindful*:ab,ti OR meditation:ab,ti OR behaviour*:ab,ti OR behavior*:ab,ti OR lifestyle*:ab,ti OR massage:ab,ti OR “foot bath”:ab,ti OR hydrotherapy:ab,ti OR cryotherap*:ab,ti OR complementary*:ab,ti OR CAM:ab,ti OR acupressure:ab,ti OR vitamin*:ab,ti OR mineral*:ab,ti OR herbal*:ab,ti OR supplement*:ab,ti OR natur*:ab,ti OR homeopath*:ab,ti OR nutraceutical*:ab,ti OR “chinese medicine*”:ab,ti OR nutrition*:ab,ti OR botanical*:ab,ti OR cannab*:ab,ti)<br>AND<br>(‘Peripheral neuropathy’/exp OR neuralgia:ab,ti OR paresthesia:ab,ti OR paraesthesia:ab,ti OR hyperalgesia:ab,ti OR CIPN:ab,ti OR neuropath*:ab,ti OR polyneuropath*:ab,ti)<br>AND<br>(‘metastasis’/exp OR ‘palliative therapy’/exp OR ‘palliative nursing’/exp OR ‘terminal care’/exp OR ‘terminally ill patient’/exp OR ‘hematologic disease’/exp OR advanced:ab,ti OR metasta*:ab,ti OR palliati*:ab,ti OR terminal:ab,ti OR "end of life":ab,ti OR incurable:ab,ti OR hospice:ab,ti OR hematolog*:ab,ti OR haematolog*:ab,ti)<br>AND<br>('randomized controlled trial'/exp OR randomized:ab,ti OR randomised:ab,ti OR RCT:ab,ti OR controlled:ab,ti)
**Web of Science**	(neoplasm* (Topic) or oncolog* (Topic) or carcinoma* (Topic) or tumour* (Topic) or tumor* (Topic) or cancer* (Topic) or malignanc* (Topic))<br>AND<br>(nonpharmacologic* (Topic) or “non pharmacologic*” (Topic) or physical* (Topic) or exercise* (Topic) or yoga (Topic) or “tai chi” (Topic) or imagery (Topic) or “art therap*” (Topic) or “mind and body” (Topic) or “mind body” (Topic) or mindful* (Topic) or meditation (Topic) or behaviour* (Topic) or behavior* (Topic) or lifestyle* (Topic) or massage (Topic) or “foot bath” (Topic) or hydrotherapy (Topic) or cryotherap* (Topic) or complementary* (Topic) or

	CAM (Topic) or acupressure (Topic) or vitamin* (Topic) or mineral* (Topic) or herbal* (Topic) or supplement* (Topic) or natur* (Topic) or homeopath* (Topic) or nutraceutical* (Topic) or “chinese medicine*" (Topic) or nutrition* (Topic) or botanical* (Topic) or cannab* (Topic))<br>AND<br>(“Peripheral Nervous System Diseases” (Topic) or neuralgia (Topic) or paresthesia (Topic) or paraesthesia (Topic) or hyperalgesia (Topic) or CIPN (Topic) or neuropath* (Topic) or polyneuropath* (Topic))<br>AND<br>(Advanced (Topic) or metasta* (Topic) or palliati* (Topic) or terminal (Topic) or "end of life" (Topic) or incurable (Topic) or OR hospice (Topic) or OR hematolog* (Topic) or haematolog* (Topic))<br>AND<br>(“randomized controlled trial” (Topic) or randomized (Topic) or randomised (Topic) or RCT (Topic) or controlled (Topic))
**CINAHL**	(MH Neoplasms OR TI neoplasm* OR AB neoplasm* OR TI oncolog* OR AB oncolog* OR TI tumour* OR AB tumour* OR TI tumor* OR AB tumor* OR TI cancer* OR AB cancer* OR TI malignanc* OR AB malignanc* OR TI carcinoma* OR AB carcinoma*)<br>AND<br>(MH “Life Style” OR MH Exercise OR MH “Physical Therapy Modalities” OR MH “Behavior Control” OR MH “Complementary Therapies” OR MH Cryotherapy OR MH “Nutrition Therapy” OR MH Balneology OR MH “Hyperthermia, Induced” OR MH “Food and Beverages” OR MH “Diet, Food, and Nutrition” OR MH “Herbal Medicine” OR MH “Plant Preparations” OR TI nonpharmacologic* OR TI “non pharmacologic*” OR TI physical* OR TI exercise* OR TI yoga OR TI “tai chi” OR TI imagery OR TI “art therap*” OR TI “mind and body” OR TI “mind body” OR TI mindful* OR TI meditation OR TI behaviour* OR TI behavior* OR TI lifestyle* OR TI massage OR TI “foot bath” OR TI hydrotherapy OR TI cryotherap* OR TI complementary* OR TI CAM OR TI acupressure OR TI vitamin* OR TI mineral* OR TI herbal* OR TI supplement* OR TI natur* OR TI homeopath* OR TI nutraceutical* OR TI “chinese medicine*” OR TI nutrition* OR TI botanical* OR TI cannab* OR AB nonpharmacologic* OR AB “non pharmacologic*” OR AB physical* OR AB exercise* OR AB yoga OR AB “tai chi” OR AB imagery OR AB “art therap*” OR AB “mind and body” OR AB “mind body” OR AB mindful* OR AB meditation OR AB behaviour* OR AB behavior* OR AB lifestyle* OR AB massage OR AB “foot bath” OR AB hydrotherapy OR AB cryotherap* OR AB complementary* OR AB CAM OR AB acupressure OR AB vitamin* OR AB mineral* OR AB herbal* OR AB supplement* OR AB natur* OR AB homeopath* OR AB nutraceutical* OR AB “chinese medicine*” OR AB nutrition* OR AB botanical* OR AB cannab*)<br>AND<br>(MH “Peripheral Nervous System Diseases” OR TI neuralgia OR AB neuralgia OR TI paresthesia OR AB paresthesia OR TI paraesthesia OR AB paraesthesia OR TI hyperalgesia OR AB hyperalgesia OR TI CIPN OR AB CIPN OR TI neuropath* OR AB neuropath* OR TI polyneuropath* OR AB polyneuropath*)

	AND<br>(MH "neoplasm metastasis" OR MH "palliative care" OR MH "terminal care" OR MH "palliative medicine" OR MH "hospice and palliative care nursing" OR MH "terminally ill" OR MH "hematologic neoplasms" OR TI advanced OR AB advanced OR TI metasta* OR AB metasta* OR TI palliati* OR AB palliati* OR TI terminal OR AB terminal OR TI "end of life" OR AB "end of life" OR TI incurable OR AB incurable OR TI hospice OR AB hospice OR TI hematolog* OR AB hematolog* OR TI haematolog* OR AB haematolog*)<br>AND<br>(PT "randomized controlled trial" OR TI randomized OR AB randomized OR TI randomised OR AB randomised OR TI RCT OR AB RCT OR TI controlled OR AB controlled)
**Cochrane CENTRAL**	#1 MeSH descriptor: [Neoplasms] explode all trees<br>#2 (neoplasm*):ti,ab,kw OR (oncolog*):ti,ab,kw OR (carcinoma*):ti,ab,kw OR (tumour*):ti,ab,kw OR (tumor*):ti,ab,kw<br>#3 (cancer*):ti,ab,kw OR (malignanc*):ti,ab,kw<br>#4 #1 OR #2 OR #3 OR #4<br>#5 MeSH descriptor: [Life Style] explode all trees<br>#6 MeSH descriptor: [Exercise] explode all trees<br>#7 MeSH descriptor: [Physical Therapy Modalities] explode all trees<br>#8 MeSH descriptor: [Behavior Control] explode all trees<br>#9 MeSH descriptor: [Complementary Therapies] explode all trees<br>#10 MeSH descriptor: [Cryotherapy] explode all trees<br>#11 MeSH descriptor: [Nutrition Therapy] explode all trees<br>#12 MeSH descriptor: [Balneology] explode all trees<br>#13 MeSH descriptor: [Hyperthermia, Induced] explode all trees<br>#14 MeSH descriptor: [Food and Beverages] explode all trees<br>#15 MeSH descriptor: [Diet, Food, and Nutrition] explode all trees<br>#16 MeSH descriptor: [Herbal Medicine] explode all trees<br>#17 MeSH descriptor: [Plant Preparations] explode all trees<br>#18 #5 OR #6 OR #7 OR #8 OR #9 OR #10 OR #11 OR #12 OR #13 OR #14 OR #15 OR #16 OR #17<br>#19 (nonpharmacologic*):ti,ab,kw OR ("non pharmacologic*"):ti,ab,kw OR (physical*):ti,ab,kw OR (exercise*):ti,ab,kw OR (yoga):ti,ab,kw<br>#20 ("tai chi"):ti,ab,kw OR (imagery):ti,ab,kw OR ("art therap*"):ti,ab,kw ("mind and body"):ti,ab,kw OR ("mind body"):ti,ab,kw<br>#21 (mindful*):ti,ab,kw OR (meditation):ti,ab,kw OR (behaviour*):ti,ab,kw OR (behavior*):ti,ab,kw<br>#22 (lifestyle*):ti,ab,kw OR (massage):ti,ab,kw OR ("foot bath"):ti,ab,kw OR (hydrotherapy):ti,ab,kw OR (cryotherap*):ti,ab,kw<br>#23 (complementary*):ti,ab,kw OR (CAM):ti,ab,kw OR (acupressure):ti,ab,kw OR (vitamin*):ti,ab,kw<br>#24 (mineral*):ti,ab,kw OR (herbal*):ti,ab,kw OR (supplement*):ti,ab,kw OR (natur*):ti,ab,kw OR (homeopath*):ti,ab,kw<br>#25 (nutraceutical*):ti,ab,kw OR ("chinese medicine*"):ti,ab,kw OR (nutrition*):ti,ab,kw OR (botanical*):ti,ab,kw OR (cannab*):ti,ab,kw

	#26 #18 OR #19 OR #20 OR #21 OR #22 OR #23 OR #24 OR #25<br>#27 MeSH descriptor: [Neoplasm Metastasis] explode all trees<br>#28 MeSH descriptor: [Palliative Care] explode all trees<br>#29 MeSH descriptor: [Terminal Care] explode all trees<br>#30 MeSH descriptor: [Palliative Medicine] explode all trees<br>#31 MeSH descriptor: [Hospice and Palliative Care Nursing] explode all trees<br>#32 MeSH descriptor: [Terminally Ill] explode all trees<br>#33 MeSH descriptor: [Hematologic Neoplasms] explode all trees<br>#34 (advanced):ti,ab,kw OR (metasta*):ti,ab,kw OR (palliati*):ti,ab,kw OR (terminal):ti,ab,kw OR ("end of life"):ti,ab,kw<br>#35 (incurable):ti,ab,kw OR (hospice):ti,ab,kw OR (hematolog*):ti,ab,kw OR (haematolog*):ti,ab,kw<br>#36 #27 OR #28 OR #29 OR #30 OR #31 OR #32 OR #33 OR #34 OR #35<br>#37 MeSH descriptor: [Peripheral Nervous System Diseases] explode all trees<br>#38 (neuralgia):ti,ab,kw OR (paresthesia):ti,ab,kw OR (paraesthesia):ti,ab,kw OR #26 (hyperalgesia):ti,ab,kw OR (CIPN):ti,ab,kw<br>#39 (neuropath*):ti,ab,kw OR (polyneuropath*):ti,ab,kw<br>#40 #37 OR #38 OR #39<br>#41 (randomized controlled trial):pt OR (randomized):ti,ab,kw OR (randomised):ti,ab,kw OR (RCT):ti,ab,kw OR (controlled):ti,ab,kw<br>#42 (control):ti,ab,kw<br>#43 #45 OR #46<br>#44 #4 AND #26 AND #36 AND #40 AND #43
**Google Scholar**	("non-pharmacological intervention" OR nutrition OR exercise OR "complementary therapies") AND<br>"chemotherapy-induced peripheral neuropathy" OR neuropathy<br>AND<br>(“advanced cancer” OR metastasis OR metastatic OR haematological OR hematological)<br>AND<br>(“randomized controlled trial” OR “randomised controlled trial” OR “randomized” or “randomised” OR controlled)

Supplementary File S2. Characteristics and findings of included studies

Supplementary Table S2. Characteristics and findings of studies that examined the effect of self-administered non-pharmacological interventions on chemotherapy-induced peripheral neuropathy symptoms and related outcomes.

**Study & Population Characteristics**				**Intervention Characteristics**		**Study Results**			
**Citation & Country**	**Population**	**Cancer**	**CTX**	**Non-pharmacological intervention**	**Control**	**CIPN Tool**	**Primary Outcome:<br>CIPN incidence/severity**	**Secondary Outcomes**	**Author conclusions**
**Physical Exercise**									
Henke et al., 2014<br><br>Country: Germany<br><br>Recruitment dates: Aug 2010-Dec 2011<br><br>Setting: inpatient<br><br>Aim: to assess the effects of a specially designed strength and endurance training on the independence and quality of life in	N: 46<br><br>Attrition: 37%<br><br>Age (yrs): >18<br><br>Females: NR<br><br>CIPN history: NR; likely some participants had pre-existing PN	Type: Lung (NSCLC or SCLC)<br><br>Advanced cancer definition: Stage IIIA/IIIB/IV; undergoing palliative CTX<br><br>Metastases: NR	Type: Platinum-based<br><br>Frequency: NR<br><br>Duration: NR<br><br>Ongoing or completed CTX: ongoing<br><br>Previous CTX: NR	Strategy: Standard care + strength and endurance training program under supervision of physiotherapist (n=25)<br><br>Regimen:<br>- 5 days per week: Endurance training (6min walking in hallway & 2min upstairs at moderate intensity) & breathing techniques (the active cycle of breathing)<br>- Every other day of the week: Strength training (4 exercises: bridging, abdominal, biceps curl, triceps extension; 3 sets, 50% of maximal amount of repetitions)<br><br>Duration: 3 CTX Cycles (from Cycle 1 Day 1 to end of Cycle 3)<br><br>Compliance: 22% withdrawn due to non-compliance	Standard care only: conventional physiotherapy (n=21)	Tool: EORTCQ LQ LC-13<br><br>Time point: Baseline & after 3 CTX Cycles	Peripheral neuropathy severity:<br>*Mean severity score (score range 0-100, higher scores indicate worse symptoms):*<br>- IG: pre: 6.25 ± 18.13; post: 20.83 ± 26.87<br>- CG: pre: 30.77 ± 37.17; post: 46.15 ± 37.36<br>- p>0.05 within groups<br>**- p=0.050** between groups	Quality of life:<br>**Global health status/quality of life:**<br>*Mean score (EORTC QLQ C-30 tool, score range 0-100, higher scores indicate higher QoL):*<br>- IG: pre: 52.08 ± 21.84; post: 57.81 ± 17.34<br>- CG: pre: 50.64 ± 28.15; post: 44.23 ± 29.54<br>- p>0.05 within and between groups<br>**Independence in carrying out activities of daily living:**<br>*Mean Barthel Index score (higher scores indicate greater independence):*<br>- IG: pre: 98.33 ± 4.20; post: 92.08 ± 15.15<br>- CG: pre: 97.78 ± 6.24; post: 81.67 ± 14.98<br>- **p<0.05** within and between groups<br><br>Pain:<br>**Overall pain:**<br>*Mean score (EORTC QLQ C-30 tool, score range 0-100, higher scores indicate worse pain):*<br>- IG: pre: 22.92 ± 30.96; post: 25.00 ± 29.19<br>- CG: pre: 51.28 ± 43.81; post: 46.15 ± 34.80<br>- p>0.05 within and between groups<br>**Pain in chest:**<br>*Mean severity score (EORTCQLQ LC-13 tool, score range 0-100, higher scores indicate worse symptoms):*<br>- IG: pre: 14.58 ± 24.25; post: 12.50 ± 26.87<br>- CG: pre: 25.64 ± 30.89; post: 17.95 ± 29.24<br>- p>0.05 within and between groups<br>**Pain in arms or shoulder:**<br>*Mean severity score (EORTCQLQ LC-13 tool, score range 0-100, higher scores indicate worse symptoms):*<br>- IG: pre: 22.92 ± 33.82; post: 10.42 ± 20.07<br>- CG: pre: 33.33 ± 38.49; post: 35.90 ± 37.17	"The training program has a positive impact on the patient's independence in carrying out activities of daily living… and endurance and strength capacity… lung cancer patients receiving a palliative chemotherapy treatment should have enhanced physical activity intervention."

lung cancer patients in stages IIIA/IIIB/IV during palliative chemotherapy<br><br>**<u>Trial registration:</u>** NR									- p>0.05 within groups<br>- **p=0.048** between groups<br>**Pain in other body parts:**<br>*Mean severity score (EORTCQLQ LC-13 tool, score range 0-100, higher scores indicate worse symptoms):*<br>- IG: pre: 31.25 ± 28.46; post: 50.00 ± 34.43<br>- CG: pre: 56.41 ± 39.40; post: 58.97 ± 38.86<br>- p>0.05 within and between groups<br><br>**<u>Physical function:</u>**<br>**Physical function:**<br>*Mean score (EORTC QLQ C-30 tool, score range 0-100, higher scores indicate higher functioning):*<br>- IG: pre: 75.42 ± 28.46; post: 74.58 ± 21.94<br>- CG: pre: 55.38 ± 29.86; post: 48.20 ± 32.90<br>- p>0.05 within groups<br>- **p=0.025** between groups<br>**Functional capacity:**<br>*6-minute walking test (meters, higher scores indicate higher functional capacity):*<br>- IG: pre: 378.35 ± 106.71; post: 397.06 ± 102.56<br>- CG: pre: 240.83 ± 150.50; post: 193.33 ± 162.78<br>- **p<0.05** within and between groups<br>*Staircase walking test (number of steps, higher scores indicate higher functional capacity):*<br>- IG: pre: 130.24 ± 51.18; post: 136.18 ± 47.53<br>- CG: pre: 70.42 ± 50.74; post: 52.08 ± 55.85<br>- **p<0.05** within and between groups<br>**Muscle strength:**<br>*Biceps curl (maximal amount of repetitions, higher scores indicate higher muscle strength):*<br>- IG: pre: 25.35 ± 10.42; post: 27.41 ± 9.75<br>- CG: pre: 14.17 ± 9.04; post: 11.75 ± 10.39<br>- **p<0.05** within and between groups<br>*Triceps extension (maximal amount of repetitions, higher scores indicate higher muscle strength):*<br>- IG: pre: 25.47 ± 10.70; post: 27.12 ± 9.81<br>- CG: pre: 16.25 ± 10.93; post: 11.08 ± 9.00<br>- **p<0.05** within and between groups<br>*Bridging (maximal amount of repetitions, higher scores indicate higher muscle strength):*<br>- IG: pre: 11.82 ± 6.88; post: 13.94 ± 6.69<br>- CG: pre: 9.25 ± 7.24; post: 7.08 ± 7.45<br>- **p<0.05** within and between groups<br>*Abdominal exercise (maximal amount of repetitions, higher scores indicate higher muscle strength):*<br>- IG: pre: 10.24 ± 5.72; post: 11.71 ± 5.67<br>- CG: pre: 6.83 ± 6.87; post: 5.00 ± 5.94<br>- **p<0.05** within and between groups<br><br>**<u>Sleep/fatigue:</u>**<br>**Fatigue:**	

								Mean score (EORTC QLQ C-30 tool, score range 0-100, higher scores indicate worse fatigue):<br>- IG: pre: 43.75 ± 28.82; post: 50.69 ± 27.51<br>- CG: pre: 59.83 ± 29.93; post: 64.10 ± 36.61<br>- p>0.05 within and between groups<br>**Insomnia:**<br>*Mean score (EORTC QLQ C-30 tool, score range 0-100, higher scores indicate worse insomnia):*<br>- IG: pre: 52.08 ± 36.45; post: 37.50 ± 34.16<br>- CG: pre: 69.23 ± 31.80; post: 53.85 ± 39.76<br>- p>0.05 within and between groups<br><br>**<u>Gastrointestinal symptoms:</u>**<br>**Nausea and vomiting:**<br>*Mean score (EORTC QLQ C-30 tool, score range 0-100, higher scores indicate worse nausea and vomiting):*<br>- IG: pre: 12.50 ± 27.55; post: 15.62 ± 22.33<br>- CG: pre: 8.97 ± 17.50; post: 32.05 ± 41.65<br>- p>0.05 within and between groups<br>**Appetite loss:**<br>*Mean score (EORTC QLQ C-30 tool, score range 0-100, higher scores indicate worse appetite loss):*<br>- IG: pre: 39.58 ± 40.77; post: 37.50 ± 41.94<br>- CG: pre: 58.97 ± 43.36; post: 58.97 ± 41.17<br>- p>0.05 within and between groups<br>**Constipation:**<br>*Mean score (EORTC QLQ C-30 tool, score range 0-100, higher scores indicate worse constipation):*<br>- IG: pre: 37.50 ± 40.14; post: 33.33 ± 32.20<br>- CG: pre: 46.15 ± 44.18; post: 46.15 ± 48.19<br>- p>0.05 within and between groups<br>**Diarrhea:**<br>*Mean score (EORTC QLQ C-30 tool, score range 0-100, higher scores indicate worse diarrhea):*<br>- IG: pre: 14.58 ± 34.36; post: 10.58 ± 34.36<br>- CG: pre: 12.82 ± 28.99; post: 17.95 ± 25.88<br>- p>0.05 within and between groups<br>**Sore mouth:**<br>*Mean severity score (EORTCQLQ LC-13 tool, score range 0-100, higher scores indicate worse symptoms):*<br>- IG: pre: 4.17 ± 11.39; post: 16.67 ± 36.51<br>- CG: pre: 17.95 ± 32.25; post: 12.82 ± 28.99<br>- p>0.05 within and between groups<br>**Dysphagia:**<br>*Mean severity score (EORTCQLQ LC-13 tool, score range 0-100, higher scores indicate worse symptoms):*<br>- IG: pre: 20.83 ± 36.26; post: 29.17 ± 38.25<br>- CG: pre: 20.51 ± 28.99; post: 25.64 ± 36.40<br>- p>0.05 within and between groups<br><br>**<u>Nutrition status:</u>** NR<br><br>**<u>Psychological outcomes:</u>**	

								**Cognitive functioning:**<br>*Mean score (EORTC QLQ C-30 tool, score range 0-100, higher scores indicate higher functioning):*<br>- IG: pre: 86.45 ± 19.45; post: 79.17 ± 31.91<br>- CG: pre: 79.49 ± 18.20; post: 57.69 ± 33.10<br>- **p=0.050** within CG; p>0.05 within IG<br>- p>0.05 between groups<br>**Emotional functioning:**<br>*Mean score (EORTC QLQ C-30 tool, score range 0-100, higher scores indicate higher functioning):*<br>- IG: pre: 69.27 ± 17.67; post: 70.31 ± 24.53<br>- CG: pre: 58.33 ± 29.27; post: 57.05 ± 28.64<br>- p>0.05 within and between groups<br><br>**Social outcomes:**<br>**Social functioning:**<br>*Mean score (EORTC QLQ C-30 tool, score range 0-100, higher scores indicate higher functioning):*<br>- IG: pre: 75.00 ± 29.19; post: 67.71 ± 29.48<br>- CG: pre: 69.23 ± 31.80; post: 60.26 ± 41.69<br>- p>0.05 within and between groups<br><br>**Treatment outcomes**: NR<br><br>**Financial:**<br>**Financial difficulties:**<br>*Mean severity score (EORTCQLQ LC-13 tool, score range 0-100, higher scores indicate worst financial difficulties):*<br>- IG: pre: 20.83 ± 34.16; post: 62.50 ± 29.50<br>- CG: pre: 25.64 ± 30.89; post: 61.54 ± 40.47<br>- **p<0.05** within groups<br>- p>0.05 between groups<br><br>**Adverse events:** NR	
Stuecher et al., 2019<br><br>**Country:** Germany<br><br>**Recruitment dates:** 2014-2016<br><br>**Setting:** outpatient, at home	**N:** 44<br><br>**Attrition:** 36%<br><br>**Age (yrs):** ≥ 50: Mean 67.1 ± 7.8 (range 50–79)<br><br>**Females:** 43%<br><br>**CIPN history:** ineligible if had	**Type:** Gastro-intestinal<br>- Pancreas (20%)<br>- Gastric (14%)<br>- Colon (52%)<br>- Esophagus (9%)<br>- NR (5%)<br><br>**Advanced Cancer Definition:** locally or systemicall	**Type:** NR<br><br>**Frequency:** NR<br><br>**Duration:** NR<br><br>**Ongoing or completed CTX:** Ongoing<br><br>**Previous CTX:** only eligible if CTX naïve	**Strategy:** Walking exercise, home-based + standard care (n=22)<br>- Individualized exercise counselling for self-managed walking by exercise specialist<br><br>**Regimen:** 150min moderate intensity walking per week<br><br>**Duration:** 12 weeks, from C1D1<br><br>**Compliance:** mean adherence rate 81%	Standard care; no walking program (n=22)	**Tool:** Rydel-Seiffer tuning fork test<br><br>**Time point:** Baseline (1 day prior to CTX C1D1), after 4-6 weeks (before CTX C3), & after 12	**Peripheral neuropathy severity:**<br>*Measured as peripheral deep sensitivity by the Rydel-Seiffer tuning fork test (measured on foot and hand, assesses ability to discriminate between different vibration intensities on scale from 0 (no sensitivity) to 8 (highest sensitivity).*<br>- no sig effects with intervention (results NR).	**Quality of life:** NR<br><br>**Pain:** NR<br><br>**Physical function:**<br>**Activities of daily living-relevant physical performance:**<br>*Mean difference in scores (measured by the short physical performance battery: combined score of gait speed, balance, and activities of daily living muscular endurance of the lower extremity; score range: 0 to 12, higher scores indicate higher functioning):*<br>- Baseline to end of intervention (12 weeks): IG: 0.4 ± 1.2, CG: 0.1 ± 2.7, p=0.36 between groups<br>**Gait speed:**<br>*Mean difference measured as Km/h:*<br>- Baseline to end of intervention (12 weeks): IG: 0.2 ± 0.5; CG: 0.2 ± 0.8, p=0.48 between groups<br>**Postural stability:**	"…It [exercise program] seems to have the potential to stabilize or improve functional capacity, postural stability and body composition. Thus, exercise might contribute to counteract treatment- and disease-related side

**Aim:** evaluate the effects of home-based moderate intensity exercise on functional capacity, activities of daily living and body composition in advanced gastrointestinal cancer patients<br><br>**Trial registration:** Clinical trials (NCT02677129)	any neurological disorder	y advanced disease stage (UICC stage III–IV)<br><br>**Metastases:** NR				weeks (end of intervention)		*Mean difference (measured by the Capacity Force-measuring Platform (CoP); measured in mm):*<br>- Baseline to end of intervention (12 weeks): IG: − 58.8 ± 139.3; CG: 58.7 ± 102.8, **p=0.003** between groups<br>**Strength of knee extensor:**<br>*Mean difference measured in N/kg/BW:*<br>- Baseline to end of intervention (12 weeks): IG: − 58.8 ± 139.3; CG: 58.7 ± 102.8, p=0.75 between groups<br><br>**Sleep/fatigue:** NR<br><br>**Gastrointestinal symptoms:** NR<br><br>**Nutrition status:**<br>**Nutrition status:**<br>Mean difference in score (measured using the Mini Nutritional Assessment (MNA):<br>- Baseline to end of intervention (12 weeks): IG: 2.2 ± 3.5; CG: −0.4 ± 5.5, **p=0.04** between groups<br>**Lean body mass:**<br>*Mean difference measured in % (measured using bioelectrical impedance analysis):*<br>- Baseline to end of intervention (12 weeks): IG: 3.4 ± 4.6; CG: 0.6 ± 3.4, **p=0.02** between groups<br><br>**Psychological outcomes:** NR<br><br>**Social outcomes:** NR<br><br>**Treatment outcomes:** NR<br><br>**Financial:** NR<br><br>**Adverse events:** None reported to be attributable to the intervention	effects and might diminish cancer- and therapy-related impairments in the physical condition and activities of daily living… No intervention-related effects on CIPN could be determined.”
Streckmann et al., 2014<br><br>**Country:** Germany<br><br>**Recruitment dates:** May 2008- Jul 2011<br><br>**Setting:** Inpatient or	**N:** 61<br><br>**Attrition:** 8%<br><br>**Age (yrs):** Adults: mean 46 (range: 19-73)<br><br>**Females:** 23%<br><br>**CIPN history:** NR	**Type:** Lymphoma<br><br>**Advanced Cancer Definition:** Participants with progressive disease<br><br>**Metastases:** NR	**Type:** NR; 61% receiving neurotoxic CTX<br><br>**Frequency:** NR<br><br>**Duration:** NR<br><br>**Ongoing or completed CTX:** Ongoing<br><br>**Previous CTX:** NR	**Strategy:** Standard care + exercise training program (n=30)<br><br>**Regimen:** One 1-hour one-on-one session twice per week, supervised by sport- or physiotherapist. Each session:<br>- Aerobic endurance training: on treadmill and/or bicycle-dynamometer<br>- Sensorimotor training: four postural stabilization tasks, 3	Standard care only, including physiotherapy (n=31)	**Tool:** Peripheral deep sensitivity evaluated by a tuning fork with a graduating scale from 0 (no sensitivity) to 8 (highest	**Peripheral neuropathy incidence:**<br>*Anytime between baseline and week 36:*<br>- IG: n=8/27 (30%), CG: n=12/27 (44%), p=0.398 between groups<br>*At week 36:* [a]<br>- IG: n=1/27 (4%), CG: n=12/27 (44%), **p=0.005**<br><br>**Peripheral deep sensitivity incidence:**<br>*Anytime between baseline and week 36:*<br>- IG: 12%, CG: 27%, **p=0.007** between groups	**Quality of life:**<br>**Health-related quality of life:**<br>*Mean (range) score change from baseline to week 36 (measured by EORTC QLQ C-30 tool, score range 0-100, higher scores indicate higher QoL):*<br>- IG: 12 (-67 to 67), p=0.033 within group; CG: -1 (-66 to 42), p=0.920 within group, p=0.113 between groups<br><br>**Pain:**<br>*Mean (range) score change from baseline to week 36 (measured by EORTC QLQ C-30 tool; higher scores indicate more pain):*<br>- IG: -16 (-100 to 67), p=0.063 within group; CG: -8 (-83 to 33), p=0.449 within groups, p=0.396 between groups<br><br>**Physical function:**<br>**Balance control:**<br>Significant differences in the change of balance control could be found between the groups, with the IG improving while the CG	“Exercise improves balance control and reduces side-effects of peripheral neuropathy, acknowledging these as substantial factors for quality of life.”

outpatient<br><br>**Aim:** assess effects of exercise in lymphoma patients during therapy<br><br>**Trial registration:** German Clinical Trials Register number: DRKS00003894.				sets, 20 seconds per set<br>- Strength training: Four resistance exercises carried out for 1-min at maximum force<br><br>**Duration:** 36 weeks<br><br>**Compliance:** 65% (for all time points and all interventions).<br>- Highest for sensorimotor training, lowest for strength<br>- Highest in stationary phases, lowest after completion of therapy		sensitivity)<br><br>**Time point:** Baseline (prior to CTX) and after 12, 24, and 36 weeks	**Peripheral deep sensitivity decline once developed:**<br>*From baseline to week 36:*<br>- IG: n=7/8 (88%), CG: n=0/12 (0%), **p<0.001** between groups<br><br>**Incidence of reduced peripheral deep sensitivity:**<br>- IG: 87.5%, CG: 0%, p<0.001 between groups	steadily declined (monopedal static ΔT3–T0; **p=0.03**; dynamic ΔT3–T0; **p=0.007**; perturbed mono-ΔT3–T0; **p=0.009** and bipedal ΔT3–T0; **p=0.006**), failed attempts (monopedal static ΔT3–T0**; p= 0.02**, dynamic ΔT3–T0; **p<0.001** and perturbed ΔT3–T0; **p=0.006**) and improved time to regain balance (ΔT3–T0; **p=0.04**).<br>**Aerobic performance and exercise carried out:**<br>The change in the aerobic performance level (ΔT3–T0; **p=0.05**) and additional amount of exercise carried out per week [metabolic equivalent (MET); **p=0.02**] differed significantly across groups.<br><br>**Sleep/fatigue:**<br>**Tiredness and sluggishness in practical tasks:**<br>*Mean score change from week 24 to week 36:*<br>- **p=0.05** between groups (results NR; p>0.05 from baseline to Wk 36)<br>**Lack of motivation and apathy:**<br>*Mean score change from baseline to week 24:*<br>- **p=0.05** between groups (results NR; p>0.05 from baseline to Wk 36)<br><br>**Gastrointestinal symptoms:**<br>**Constipation:**<br>*Mean (range) score change from baseline to week 36 (measured by EORTC QLQ C-30 tool, score range 0-100, higher scores indicate more constipation):*<br>- IG: -19 (-100 to 33), p=0.047 within group; CG: -11 (-67 to 33), p=0.117 within group; p=0.537 between groups<br>**Diarrhea:**<br>*Mean (range) score change from baseline to week 24 (measured by EORTC QLQ C-30 tool, score range 0-100, higher scores indicate more diarrhea):*<br>- IG: 18 (-100 to 100), p=0.016 within group; CG: -6 (-100 to 100), p=1.000 within group, **p=0.039** between groups<br>between groups<br><br>**Nutrition status:** NR<br><br>**Psychological outcomes:**<br>**Emotional function:**<br>*Mean (range) score change from week 24 to week 36 (measured by EORTC QLQ C-30 tool, score range 0-100, higher scores indicate more constipation):*<br>- IG: 10 (-17 to 42); CG: -4 (-42 to 50), **p=0.007** between groups<br><br>**Social outcomes:** NR<br><br>**Treatment outcomes:** NR<br><br>**Financial:**<br>**Financial problems:**	

								Mean (range) score change from week 24 to week 36 (measured by EORTC QLQ C-30 tool, score range 0-100, higher scores indicate more constipation):<br>- IG: -5 (-33 to 67); CG: 4 (0 to 33), **p=0.045** between groups<br><br>**Adverse events:**<br>No serious adverse events reported.<br>**Side effects (unspecified):**<br>*Mean (range) change from baseline to week 36:*<br>- IG: -2.1 (-16 to 6), CG: -0.4 (-6 to 5), p=0.226 between groups	
Zimmer et al., 2018<br><br>**Country:** Germany<br><br>**Recruitment dates:** Mar 2015- Jan 2016<br><br>**Setting:** Outpatient; sports center<br><br>**Aim:** the influence of a supervised exercise program on CIPN in metastasized colorectal cancer<br><br>**Trial registration:** NR	**N:** 30<br><br>**Attrition:** 20%<br><br>**Age (yrs):** Adults: mean: IG: 69, CG: 70 (range 50-81)<br><br>**Females:** 30%<br><br>**CIPN history:** NR; likely some had existing CIPN	**Type:** Colorectal<br>- cecum/ colon (57%)<br>- rectosigmoid junction (10%)<br>- rectum (33%)<br><br>**Advanced Cancer Definition:** metastasized, stage IV<br><br>**Metastases:** 100% yes<br>- Liver (77%)<br>- Lung (47%)<br>- Peritoneum (23%)<br>- Bone (3%)<br>- Other (10%)	**Type**: mixed<br><br>**Frequency:** NR<br><br>**Duration:** mean number of cycles:<br>- During study period: IG: 2, CG: 3<br>- Prior to baseline: IG: 28, CG: 24<br><br>**Ongoing or completed CTX:** both; 33% had no CTX during study period<br><br>**Previous CTX:** 63% had received oxaliplatin before baseline	**Strategy:** Exercise program, supervised by sports therapist (n=17)<br><br>**Regimen:** Two 60-minute sessions per week<br>- Balance 10min<br>- Coordination 5min<br>- Endurance 10min<br>- Resistance 20min<br>- Cool down 15min<br><br>**Duration:** 8 weeks<br><br>**Compliance:** 80%	Standard care; written standard recommendations to obtain physical fitness (n=13)	**Tool:** TOI of FACT/GOG-NTX<br><br>**Time point:** at baseline (before intervention), post-intervention (8 weeks post baseline), and 4-weeks post-intervention (12 weeks post baseline)	**CIPN symptom severity:**<br>*Mean score (TOI of FACT/GOG-NTX [combined score of the physical well-being, functional well-being, and neurotoxicity subscale], score range: 0-100, higher scores indicate less symptoms):*<br>- Baseline: IG: 75.1 ± 14.8; CG: 71.6 ± 13.0<br>- Post-intervention: IG: 77.4 ± 11.8; CG: 64.4 ± 11.6<br>- 4 weeks post-intervention: IG: 75.4 ± 13.6; CG: 63.5 ± 9.8<br>- Baseline to post-intervention p between groups: **p=0.028**<br>- Baseline to 4-weeks post-intervention p between groups: **p=0.031**<br>- Post-intervention to 4-weeks post-intervention p between groups: p=0.592<br><br>**Neurotoxicity symptom severity:**<br>*Mean score (neurotoxicity subscale of FACT-G/GOG-NTX, score range: 0-44, higher scores indicate less symptoms):*<br>- Baseline: IG: 33.1 ± 8.2; CG: 34.1 ± 7.3<br>- Post-intervention: IG: 35.2 ± 6.6; CG: 30.0 ± 9.9<br>- 4 weeks post-intervention: IG: 34.0 ± 7.4; CG: 29.4 ± 9.3<br>- Baseline to post-intervention p between groups: **p=0.002**<br>- Baseline to 4-weeks post-intervention p between groups: **p=0.015**<br>- Post-intervention to 4-weeks post-intervention p between groups: p=0.385	**Quality of life:**<br>**Health-related quality of life:**<br>*Mean score (total FACT-G/GOG-NTX, score range: 0-108, higher scores indicate better quality of life):*<br>- Baseline: IG: 82.4 ± 15.5; CG: 0.5 ± 12.8<br>- Post-intervention: IG: 82.6 ± 15.4; CG: 77.9 ± 12.0<br>- 4 weeks post-intervention: IG: 81.8 ± 15.4; CG: 75.9 ± 10.5<br>- Baseline to post-intervention p between groups: p=0.303<br>- Baseline to 4-weeks post-intervention p between groups: p=0.283<br>- Post-intervention to 4-weeks post-intervention p between groups: p=0.805<br><br>**Pain:** NR<br><br>**Physical function:**<br>**Physical wellbeing:**<br>*Mean score (subscale of FACT-G/GOG-NTX, score range: 0-28, higher scores indicate better wellbeing):*<br>- Baseline: IG: 22.9 ± 3.8; CG: 19.6 ± 5.4<br>- Post-intervention: IG: 23.2 ± 3.8; CG: 19.4 ± 3.7<br>- 4-weeks post-intervention: IG: 22.3 ± 4.3; CG: 18.1 ± 3.5<br>- Baseline to post-intervention p between groups: p=0.742<br>- Baseline to 4-weeks post-intervention p between groups: p=0.509<br>- Post-intervention to 4-weeks post-intervention p between groups: p=0.650<br>**Functional wellbeing:**<br>*Mean score (subscale of FACT-G/GOG-NTX, score range: 0-28, higher scores indicate better wellbeing):*<br>- Baseline: IG: 19.0 ± 5.3; CG: 17.9 ± 6.2<br>- Post-intervention: IG: 18.9 ± 6.0; CG: 16.1 ± 6.2<br>- 4-weeks post-intervention: IG: 19.1 ± 5.4; CG: 16.0 ± 5.7<br>- Baseline to post-intervention p between groups: p=0.094<br>- Baseline to 4-weeks post-intervention p between groups: p=0.123<br>- Post-intervention to 4-weeks post-intervention p between groups: p=0.742<br>**Balance:**<br>*Mean score (measured by total GGT-Reha; higher score indicates better balance)*<br>- Baseline: IG: 54.9 ± 16.7; CG: 44.0 ± 16.3<br>- Post-intervention: IG: 58.9 ± 12.8 ; CG: 47.2 ± 12.8	"In conclusion, this study provides first evidence that a multi- modal exercise program counteracts a worsening of CIPN and further improves balance and strength in a palliative setting with patients suffering from metastatic colorectal cancer."

								- 4-weeks post-intervention: IG: 61.4 ± 13.5; CG: 46.4 ± 13.2<br>- Baseline to post-intervention p between groups: p=0.934<br>- Baseline to 4-weeks post-intervention p between groups: p=0.650<br>- Post-intervention to 4-weeks post-intervention p between groups: p=0.145<br>**Endurance capacity:**<br>*6-minute walk test (meters walked in 6 minutes, higher value indicates greater endurance capacity):*<br>- Baseline: IG: 477.7 ± 91.9; CG: 459.7 ± 74.1<br>- Post-intervention: IG: 502.2 ± 62.1; CG: 478.2 ± 75.2<br>- 4-weeks post-intervention: IG: 519.1 ± 69.0; CG: 482.2 ± 82.6<br>- Baseline to post-intervention p between groups: p=1.000<br>- Baseline to 4-weeks post-intervention p between groups: p=0.432<br>- Post-intervention to 4-weeks post-intervention p between groups: p=0.509<br>**Dynamic muscular strength:**<br>*Hypothetic one-repetition maximum (h1RM; measured in kilograms; h1RM = percentage of maximum power x 100%; higher h1RM indicates higher strength:*<br>*Bench press:*<br>- Baseline: IG: 38.1 ± 12.8; CG: 40.2 ± 19.1<br>- Post-intervention: IG: 49.1 ± 23.8; CG: 38.2 ± 15.0<br>- 4-weeks post-intervention: IG: 46.4 ± 24.2; CG: 36.4 ± 12.6<br>- Baseline to post-intervention p between groups: **p=0.014**<br>- Baseline to 4-weeks post-intervention p between groups: **p=0.014**<br>- Post-intervention to 4-weeks post-intervention p between groups: p=0.536<br>*Leg press:*<br>- Baseline: IG: 142.2 ± 44.1; CG: 166.7 ± 56.3<br>- Post-intervention: IG: 180.9 ± 66.6; CG: 160.3 ± 54.1<br>- 4-weeks post-intervention: IG: 179.7 ± 68.2; CG: 160.0 ± 62.2<br>- Baseline to post-intervention p between groups: **p=0.001**<br>- Baseline to 4-weeks post-intervention p between groups: **p=0.011**<br>- Post-intervention to 4-weeks post-intervention p between groups: p=0.845<br>*Lat pulldown:*<br>- Baseline: IG: 29.7 ± 9.1; CG: 30.5 ± 10.3<br>- Post-intervention: IG: 38.4 ± 14.0; CG: 32.9 ± 11.3<br>- 4-weeks post-intervention: IG: 36.4 ± 13.3; CG: 31.2 ± 11.6<br>- Baseline to post-intervention p between groups: **p=0.022**<br>- Baseline to 4-weeks post-intervention p between groups: **p=0.031**<br>- Post-intervention to 4-weeks post-intervention p between groups: p=0.837<br><br>**Sleep/fatigue:** NR<br><br>**Gastrointestinal symptoms:** NR	

								**Nutrition status:** NR<br><br>**Psychological outcomes:**<br>**Emotional wellbeing:**<br>*Mean score (subscale of FACT-G/GOG-NTX, score range: 0-24, higher scores indicate better wellbeing):*<br>- Baseline: IG: 17.4 ± 5.1; CG: 18.1 ± 3.7<br>- Post-intervention: IG: 17.6 ± 4.8; CG: 18.3 ± 4.2<br>- 4-weeks post-intervention: IG: 17.6 ± 4.4; CG: 18.2 ± 3.8<br>- Baseline to post-intervention p between groups: p=1.000<br>- Baseline to 4-weeks post-intervention p between groups: p=0.408<br>- Post-intervention to 4-weeks post-intervention p between groups: p=0.536<br><br>**Social outcomes:**<br>**Social wellbeing:**<br>*Mean score (subscale of FACT-G/GOG-NTX, score range 0-28, higher scores indicate better wellbeing):*<br>- Baseline: IG: 23.1 ± 3.8; CG: 24.2 ± 2.5<br>- Post-intervention: IG: 22.9 ± 3.9; CG: 24.1 ± 3.0<br>- 4-weeks post-intervention: IG: 22.7 ± 4.2; CG: 23.6 ± 2.9<br>- Baseline to post-intervention p between groups: p=0.616<br>- Baseline to 4-weeks post-intervention p between groups: p=0.984<br>- Post-intervention to 4-weeks post-intervention p between groups: p=0.509<br><br>**Treatment outcomes:** NR<br><br>**Financial:** NR<br><br>**Adverse events:**<br>- No serious adverse events observed	
**Nutrition supplements**									
Bradfield et al., 2015<br><br>**Country:** USA<br><br>**Recruitment dates:** Oct 2007-Sep 2012	**N:** 200<br><br>**Attrition:** 23%<br><br>**Age (yrs):** Mean:<br>- IG: 8.4 ± 4.7<br>- CG: 8.5 ± 4.8<br><br>**Females:** 38%	**Type:** ALL (n=179), NHL (n=20)<br><br>**Advanced Cancer Definition:** Hematological<br><br>**Metastases:** NR	**Type:** Vincristine + steroid<br><br>**Frequency:** weekly<br><br>**Duration:** at least 4 weeks, starting from Cycle 1<br><br>**Ongoing or completed CTX:** Ongoing<br><br>**Previous CTX:** NR	**Strategy:** L-glutamic acid in capsule form (n=101)<br><br>**Regimen:** 250mg capsules (1 capsule for body surface area <1m2 and 2 capsules for ≥1m2), three times daily<br><br>**Duration:** 5 weeks, starting pre-CTX to 7-days post week 4 dose of CTX	Placebo (n=99), based on body weight as per IG	**Tool:** Modified Balis Pediatric Scale of Peripheral Neuropathies<br><br>**Time point:** At baseline (before CTX) and post-	**Neurotoxicity incidence:**<br>*Grade ≥2:*<br>- IG: n=21/79 (27%)<br>- CG: n=25/88 (28%)<br>- p between groups: p=0.863	**Quality of life:** NR<br><br>**Pain:** NR<br><br>**Physical function:** NR<br><br>**Sleep/fatigue:** NR<br><br>**Gastrointestinal symptoms:** NR<br><br>**Nutrition status:** NR<br><br>**Psychological outcomes:** NR<br><br>**Social outcomes:** NR	"Glutamic acid is not effective in the pre-adolescent population for prevention, and alternative methods of supportive care will need to be investigated."<br><br>***Stratum 2 only eligible, not Stratum 1**

**Setting**: Outpatient<br><br>**Aim:** To determine if there is protection against neurotoxicity with the administration of glutamic acid in pediatric cancer patients receiving vincristine therapy.<br><br>**Trial registration:** NR	**CIPN** history: ineligible if neuromuscular disease or abnormal baseline neurologic exam			**Compliance:** Patients who received full dose: IG: 93%; CG: 97%		intervention (at 5 weeks)		**Treatment outcomes:** NR<br><br>**Financial:** NR<br><br>**Adverse events:** NR	**ineligible as Wilms tumor or rhabdomyosarcoma not advanced/ hematological**
Howells et al., 2019<br><br>**Country:** UK<br><br>**Recruitment dates:** Apr 2013-May 2016<br><br>**Setting**: Outpatient<br><br>**Aim:** to assess safety, efficacy, quality of	**N:** 27<br><br>**Attrition**: 22%<br><br>**Age (yrs):** Adults: Mean 67.6 (range 53-78)<br><br>**Females**: NR<br><br>**CIPN history:** Ineligible if existing of CIPN	**Type:** Colorectal<br><br>**Advanced Cancer Definition:** Metastatic, stage M1a-M1c<br><br>**Metastases:**<br>- Liver (96%)<br>- Lung (48%)<br>- Peritoneal (15%)<br>- Adrenal (7%)<br>-Portal hepatic nodes (4%)	**Type**: Folinic acid/ 5-fluorouracil/ oxaliplatin ± bevacizumab<br><br>**Frequency:** Once every two weeks<br><br>**Duration:** ≤12 cycles or until patient progression, unacceptable toxicity, death, or withdrawal<br><br>**Ongoing or completed CTX:** Ongoing<br><br>**Previous CTX:** ineligible if had undergone CTX (other than adjuvant for colorectal cancer)	**Strategy:** Standard care + oral curcumin powder in capsule form (C3 Complex; 80% Curcumin & 20% curcuminoids (demethoxycurcumin/ bisdemethoxycurcumin)) (n=18)<br><br>**Regimen:** total of 2g per day (four 0.5g capsules per day)<br><br>**Duration:** from 1 week prior to commencing CTX to the cessation of CTX<br><br>**Compliance:**<br>- 4% excluded as took capsules <80% of the time<br>- Curcumin glucuronide detectable	Standard care only (n=9)	**Tool:** EORTC-QLQ-C30 / NCI-CTAE<br><br>**Time point:** baseline (pre CTX) and when CTX ceased (i.e., end of trial)	**Peripheral neuropathy severity:**<br>- No between group differences (data NR); p (at 6 Cycles) =0.223; p (at 12 Cycles) =0.204)<br><br>**Peripheral neuropathy Incidence:** [a]<br>*Incidence of adverse event (measured using the NCI-CTAE).*<br>- *Any grade:* IG: n=16/18 (89%), CG: n=4/9 (44%), **p=0.02**<br>- *Grade 1-2:* IG: n=16/18 (89%), CG: n=4/9 (44%), **p=0.02**<br>- *Grade 3-4:* IG: n=1/18 (6%), CG: n=0/9 (0%), p=0.77	**Quality of life:**<br>*Mean EORTC-QLQ30 percentage score (higher score indicates better quality of life):*<br>- Baseline: IG: 70%, CG: 65%<br>- End of trial: IG: 60%, CG: 45%<br>- p between groups: p=0.248<br><br>**Pain:** [a]<br>*Measured using the NCI-CTAE.*<br>*Incidence of adverse events possibly or probably attributable to curcumin (occurring in ≥2.5% of patients):*<br>- *Abdominal pain:* IG: n=2/18 (11%) vs. CG: n=0/9 (0%), p=0.51<br><br>**Physical function:**<br>**Physical function:** [a]<br>*Mean EORTC-QLQ30 percentage score (higher score indicates better functioning):*<br>- Baseline: IG: 80%, CG: 75%<br>- End of trial: IG: n=13/18 (70%), CG: n=6/9 (65%), p=0.77<br>**Symptom score:**<br>*Mean EORTC-QLQ30 percentage score (higher score indicates worse symptoms):*<br>- Baseline: IG: 20%, CG: 25%<br>- End of trial: IG: n=4/18 (24%), CG: n=3/9 (29%), p=0.54	"Curcumin is a safe and tolerable adjunct to CTX in patients with metastatic colorectal cancer… No significant differences were reported, although slightly higher neuropathy scores were observed in CG than in IG patients and so may warrant further exploration in future studies."

life, and neurotoxicity of curcumin in patients receiving CTX<br><br>**<u>Trial registration:</u>**<br>Clinical Trials Register (NCT01490996)				at concentrations >1.00 pmol/mL in 83%				**<u>Sleep/fatigue:</u>**<br>**Insomnia:** [a]<br>*Incidence of adverse event (measured using the NCI-CTAE).*<br>- *Any grade (all were grade 1-2):* IG: n=3/18 (17%), CG: n=0/9 (0%), p=0.35<br>**Fatigue:** [a]<br>*Incidence of adverse event (measured using the NCI-CTAE).*<br>- *Any grade (all were grade 1-2):* IG: n=15/18 (83%), CG: n=5/9 (56%), p=0.13<br><br>**<u>Gastrointestinal symptoms:</u>** [a]<br>*Measured using the NCI-CTAE.*<br>*Incidence of adverse events possibly or probably attributable to curcumin (occurring in ≥2.5% of patients):*<br>- *Anorexia:* IG: n=4 (22%) vs. CG: n=0, p=0.25<br>- *Bloating:* IG: n=2 (11%) vs. CG: n=0, p=0.51<br>- *Constipation:* IG: n=5 (28%) vs. CG: n=0, p=0.18<br>- *Diarrhea:* IG: n=10 (56%) vs. CG: n=0, **p=0.04**<br>- *Dry mouth:* IG: n=2 (11%) vs. CG: n=0, p=0.51<br>- *Dyspepsia:* IG: n=7 (39%) vs. CG: n=0, p=0.10<br>- *Flatulence:* IG: n=2 (11%) vs. CG: n=0, p=0.51<br>- *Nausea:* IG: n=8 (44%) vs. CG: n=0, p=0.07<br>- *Oral mucositis:* IG: n=6 (33%) vs. CG: n=0, p=0.13<br>- *Vomiting:* IG: n=5 (28%) vs. CG: n=0, p=0.18<br><br>**<u>Nutrition status:</u>**<br>**Weight loss:**<br>*Incidence of adverse event (measured using the NCI-CTAE).*<br>- *Any grade:* IG: n=3/18 (17%), CG: n=2/9 (22%), p=0.73<br><br>**<u>Psychological outcomes:</u>** NR<br><br>**<u>Social outcomes:</u>** NR<br><br>**<u>Treatment outcomes:</u>**<br>**Progression-free survival:**<br>*Median (range) days:*<br>- IG: 320 (175-405), CG: 171 (9-214), p NR<br>- HR: 0.57 (95% CI: 0.24-1.36, p=0.200)<br>**Overall survival:**<br>*Median (range) days:*<br>- IG: 596 (323-still alive), CG: 200 (9-563), p NR<br>- HR: 0.34 (95% CI: 0.14-0.82, **p=0.016**)<br>**Objective response rate:**<br>*Incidence at Cycle 6:*<br>- IG: 67%; CG: 44%, p=0.285 between groups<br>*Incidence at Cycle 12:*<br>- IG: 53%; CG: 11%, **p=0.039** between groups<br>**Complete response rate:**<br>*Incidence 1-3 months post baseline:*<br>- IG: 0%; CG: 0%<br>**Stable disease:**<br>*Incidence 1-3 months post baseline:*	

								- IG: 28%; CG: 22%; p=0.756 between groups<br>**Partial response:**<br>*Incidence 1-3 months post baseline:*<br>- IG: 56%; CG: 44%; p=0.586 between groups<br><br>**Financial:** NR<br><br>**Adverse events:**<br>*Measured using the NCI-CTAE*<br>*Incidence of adverse events possibly or probably attributable to curcumin (occurring in ≥2.5% of patients):*<br>- *Acute kidney injury:* IG: n=2 (11%) vs. CG: n=0, p=0.51	
Sanchez-Lara et al., 2014<br><br>**Country:** Mexico<br><br>**Recruitment dates:** Feb 2010-Aug 2012<br><br>**Setting**: Outpatient<br><br>**Aim:** To compare the effect of an oral EPA enriched supplement with an isocaloric diet on nutri-tional, clinical and inflammatory parameters and HRQL in advanced	**N:** 112<br><br>**Attrition:** 25%<br><br>**Age (yrs):** 18-80 years<br><br>**Females:** 53%<br><br>**CIPN history:** NR	**Type:** non-small cell lung<br><br>**Advanced Cancer Definition:** Stage IIIb (22%) and IV (78%)<br><br>**Metastases:** NR	**Type**: paclitaxel (175 mg/m^2) and cisplatin (75mg/m^2)/ carboplatin<br><br>**Frequency:** every 3 weeks<br><br>**Duration:** at least 2 cycles and maximum of 6 cycles<br><br>**Ongoing or completed CTX:** Ongoing<br><br>**Previous CTX:** no prior cytotoxic CTX	**Strategy:** omega 3 (eicosapentaenoic acid)-enriched oral nutrition supplement + isocaloric diet (n=54)<br>- provided in drink (ProSure, Abbott Nutrition)<br>- Also contains protein, dietary fiber, 28 vitamins & minerals (incl. B6, B12, A, E), fatty acids, amino acids.<br>- isocaloric diet based on individual energy requirements, advised to follow a diet based on standardized menus of 1400, 1600, 1800, 2000 or 2200 kcal. Energy in drinks (590 kcal) was subtracted, so no extra calories were provided.<br><br>**Regimen:** 2 237mL drinks per day, provides 2.2g eicosapentaenoic acid daily<br><br>**Duration:** 2 CTX cycles, from C1D1<br><br>**Compliance:** NR	Isocaloric diet; advised to follow standardized menus as per IG (n=58)	**Tool:** EORTC-QLQ-C30/-LC13<br><br>**Time point:** Baseline (prior to CTX), 7-days post CTX C1, & 7 days post CTX C2)	**Neuropathy severity:**<br>*Mean (higher scores indicate worse neuropathy):*<br>- Baseline: IG: 19.9 ± 29; CG: 11.7 ± 22<br>- Post-C2: IG: 20.9 ± 25; CG: 31.8 ± 30<br>- Baseline to post-C2 p between groups: **p=0.05**	**Quality of life:**<br>**Global health status:**<br>*Mean (measured by EORTC-QLQ- C30 and QLQ-LC13, higher scores indicate better health status):*<br>- Baseline: IG: 54.3 ± 28; CG: 62.3 ± 23<br>- Post-C2: IG: 65.4 ± 23; CG: 56.5 ± 26<br>- Baseline to post-C2 p between groups: p=0.136<br><br>**Pain:** NR<br><br>**Physical function:**<br>**Physical functioning:**<br>*Mean (measured by EORTC-QLQ- C30 and QLQ-LC13, higher scores indicate better functioning):*<br>- Baseline: IG: 64.5 ± 31; CG: 74.0 ± 23<br>- Post-C2: IG: 72.5 ± 22; CG: 72.3 ± 27<br>- Baseline to post-C2 p between groups: p=0.419<br><br>**Sleep/fatigue:**<br>**Fatigue severity:**<br>*Mean (measured by EORTC-QLQ- C30 and QLQ-LC13, higher scores indicate worse fatigue):*<br>- Baseline: IG: 42.7 ± 24; CG: 35.9 ± 21<br>- Post-C2: IG: 32.3 ± 24; CG: 34.7 ± 20<br>- Baseline to post-C2 p between groups: **p=0.04**<br><br>**Gastrointestinal symptoms:**<br>**Appetite loss:**<br>*Mean (measured by EORTC-QLQ- C30 and QLQ-LC13, higher scores indicate worse appetite loss):*<br>- Baseline: IG: 41.5 ± 34; CG: 36.8 ± 34<br>- Post-C2: IG: 34.9 ± 31; CG: 28.2 ± 30<br>- Baseline to post-C2 p between groups: **p=0.05**<br>**Nausea and vomiting:**<br>*Mean (measured by EORTC-QLQ- C30 and QLQ-LC13, higher scores indicate worse nausea and vomiting):*<br>- Baseline: IG: 19.5 ± 23; CG: 8.2 ± 14<br>- Post-C2: IG: 27.9 ± 30; CG: 19.6 ± 21<br>- Baseline to post-C2 p between groups: p=0.830<br>**Diarrhea:**	"Administration of ONS-EPA seems effective in improving the nutritional status (including lean body mass), as well as energy and protein intake and decreased fatigue and neuropathy in non-small cell lung cancer patients undergoing CTX."

NSCLC patients<br><br>**<u>Trial registration:</u>**<br>Clinical trials (NCT01048970)								*Mean (measured by EORTC-QLQ- C30 and QLQ-LC13, higher scores indicate worse diarrhea):*<br>- Baseline: IG: 20.0 ± 27; CG: 6.1 ± 13<br>- Post-C2: IG: 12.0 ± 12; CG: 8.6 ± 14<br>- Baseline to post-C2 p between groups: p=0.19<br><br>**<u>Nutrition status:</u>**<br>**Body weight:**<br>*Mean (reported in kg):*<br>- Baseline: IG: 60.4 ± 11; CG: 64.7 ± 13<br>- Post-C2: IG: 60.1 ± 11; CG: 62.6 ± 14<br>- Baseline to post-C2 p between groups: **p=0.01**<br>**Lean body mass:**<br>*Mean (reported in kg; measured using bioimpedance):*<br>- Baseline: IG: 36.2 ± 10; CG: 43.9 ± 14<br>- Post-C2: IG: 37.8 ± 9; CG: 42.0 ± 13<br>- Baseline to post-C2 p between groups: **p=0.01**<br><br>**<u>Psychological outcomes:</u>** NR<br><br>**<u>Social outcomes:</u>** NR<br><br>**<u>Treatment outcomes:</u>**<br>**Tumour response rate:**<br>*Mean %*<br>- IG: 47.5% (95% CI: 33-61.9); CG: 46.3% (95% CI: 31.9-60.7), p=0.92 between groups<br>**Incidence of stable disease:**<br>*% with stable disease:*<br>- IG: 47.6% (95% CI: 36.2-62); CG: 35.9% (95% CI: 22-49.8); p=NR<br>**Overall survival:**<br>*Median months:*<br>- IG: 14.9 (95% CI: 8.8-21.1); CG: 12.1 (95% CI: 10.1-14.2), p=0.94<br>**Progression-free survival:**<br>*Median months:*<br>- IG: 7.6 (95% CI: 6.3-8.9), CG: 6.3 (95% CI: 5.1-7.4), p NR<br><br>**<u>Financial:</u>** NR<br><br>**<u>Adverse events:</u>**<br>*Measured by CTCAE:*<br>- No between group differences found in biochemical and hematological toxicity (data NR).	
Wang et al., 2007<br><br>**<u>Country:</u>**<br>Taiwan<br><br>**<u>Recruitment dates:</u>**	**<u>N:</u>** 86<br><br>**<u>Attrition</u>:**<br>NR<br><br>**<u>Age (yrs):</u>**<br>Adults:	**<u>Type:</u>**<br>Colorectal<br>- Colon (66%)<br>- Rectum (34%)<br><br>**<u>Advanced Cancer</u>**	**<u>Type</u>:** Oxaliplatin (85mg/m2) & FA 20mg/m2 day 1 and day 15; 5FU 500mg/m2 days 1, 8, 15<br><br>**<u>Frequency:</u>** every 4 weeks	**<u>Strategy:</u>** Levo-Glutamine (n=42)<br><br>**<u>Regimen:</u>** 30g total (15g twice daily)<br><br>**<u>Duration:</u>** 6 cycles; 7 days, every 2 weeks starting on the day of	Standard care (n=44)	**<u>Tool:</u>**<br>NCI-CTC & when possible electrophysiological examinations,	**<u>PN incidence:</u>**<br>**Grade 0 (no) PN:** [a]<br>- *After 2 cycles:* IG: n=35/42 (83%), CG: n=26/44 (59%), **p=0.02**<br>- *After 4 cycles:* IG: n=29/42 (69%), CG: n=26/44 (59%), p=0.34	**<u>Quality of life:</u>**<br>**Incidence of interference with activities of daily living:**<br>- IG: n=7/42 (17%), CG: n=18/44 (41%), **p=0.02**<br><br>**<u>Pain:</u>** NR<br><br>**<u>Physical function:</u>** NR<br><br>**<u>Sleep/fatigue:</u>** NR	"Oral glutamine has a potential neuroprotective effect in metastatic colorectal cancer patients treated

Sep 2004-Dec 2005<br><br>**Setting:** Outpatient<br><br>**Aim:** to assess the efficacy of glutamine in preventing oxaliplatin-induced neuropathy<br><br>**Trial registration:** NR	- ≥50 (60%)<br>- <50 (40%)<br><br>**Females:** 35%<br><br>**CIPN history:** Ineligible if had neuropathy	**Definition:** Metastatic<br><br>**Metastases:**<br>- Liver (40%)<br>- Lung (23%)<br>- Liver and lung (23%)<br>- Other (14%)	**Duration:** NR<br><br>**Ongoing or completed CTX:** Ongoing<br>- If grade 3–4 PN: oxaliplatin dose reduced by 25% of the previous dose until recovery<br>- If intolerable PN or persistent functional impairment, oxaliplatin omitted from CTX regimen.<br><br>**Previous CTX:** no previous therapy for metastatic diseases (adjuvant therapy was allowed if more than 6 months had transpired since its completion).	oxaliplatin treatment (C1D1)<br><br>**Compliance:** NR		including SAP, NCV, CMAP, F wave latency<br><br>**Time point:** at Baseline (before CTX), and after 2, 4, 6 CTX cycles	- *After 6 cycles:* IG: n=20/42 (48%), CG: n=12/44 (27%), p=0.05<br>**Grade 1-2 PN:**<br>- *After 2 cycles:* IG: n=7/42 (17%), CG: n=17/44 (39%), **p=0.04**<br>- *After 4 cycles:* [a] IG: n=11/42 (26%), CG: n=16/44 (36%), p=0.31<br>- *After 6 cycles:* [a] IG: n=17/42 (41%), CG: n=18/44 (41%), p=0.97<br>**Grade 3-4 PN:**<br>- *After 2 cycles: a* IG: n=0/42 (0%), CG: n=1/44 (2%), p=0.51<br>- *After 4 cycles:* IG: n=2/42 (5%), CG: n=8/44 (18%), **p=0.05**<br>- *After 6 cycles:* IG: n=5/42 (12%), CG: n=14/44 (32%), **p=0.04**<br><br>**Acute, cold-induced PN incidence:**<br>- IG: n=14/42 (33%), CG: n=25/44 (57%), **p=0.03**<br><br>**Abnormal electrophysiological examination:**<br>*Carried out in n=28 who experienced grade 1–4 PN (IG: n=14, CG: n=14)*<br>- IG: n=9/14 (64%), CG: n=11/14 (79%), p=0.68	**Gastrointestinal symptoms:** NR<br><br>**Nutrition status:** NR<br><br>**Psychological outcomes:** NR<br><br>**Social outcomes:** NR<br><br>**Treatment outcomes:**<br>**Incidence of oxaliplatin dose reduction:**<br>- IG: n=3/42 (7%), CG: n=12/44 (27%), **p=0.02**<br>**Survival:**<br>- Incidence of survival ≥12 months: IG: n=30/42 (71%), CG: n=35/44 (80%), p=0.46<br>- Median survival time: 17.3 months vs. 18.6 months (groups not specified), p=0.79<br>**Response to oxaliplatin-based CTX:**<br>*Assessed using World Health Organization criteria:*<br>*- Complete remission:* IG: n=5/42 (12%), CG: n=3/44 (7%), p NR<br>*- Partial remission:* IG: n=17/42 (41%), CG: n=18/44 (41%), p NR<br>*- Overall response:* IG: n=22/42 (52%), CG: n=21/44 (48%), p=0.90<br>*- Stable disease:* IG: n=12/42 (29%), CG: n=13/44 (30%), p NR<br>*- Progressive disease:* IG: n=8/42 (19%), CG: n=10/44 (23%), p NR<br><br>**Financial:** NR<br><br>**Adverse events:**<br>**Incidence of grade 3-4 non-neurological toxicities:**<br>*Assessed using World Health Organization criteria:*<br>- *Leukopenia:* IG: n=4/42 (10%), CG: n=5/44 (11%), p=0.78<br>- *Thrombocytopenia:* IG: n=5/42 (12%), CG: n=4/44 (9%), p=0.67<br>- *Elevated liver enzymes* IG: n=1/42 (2%), CG: n=1/44 (2%), p=0.97<br>- *Impaired renal function:* IG: n=1/42 (2%), CG: n=0/44 (0%), p=0.48	with oxaliplatin, and may therefore improve the therapeutic index … Although glutamine supplementation significantly reduced the incidence of "subjective" neuropathy in these patients, it did not exert a protective effect on the deterioration of electrophysiological tests."
**Japanese herbal medicine**									
Motoo et al., 2020<br><br>**Country:** Japan<br><br>**Recruitment dates:** Mar 2014-Oct 2017	**N:** 52<br><br>**Attrition:** 23%<br><br>**Age (yrs):** Adults: median: IG: 62, CG: 68 (range 35-79)	**Type:** Colorectal<br>- Colon (58%)<br>- Rectum (42%)<br><br>**Advanced Cancer Definition:** Stage 3 (IIIa 40%, IIIb 60%)	**Type:** capecitabine (2400 mg/m2) and oxaliplatin (130 mg/m2)<br><br>**Frequency:** every 3 weeks<br><br>**Duration:** 8 postoperative CTX cycles<br><br>**Ongoing or completed CTX:** ongoing	**Strategy:** ninjin'yoeito powder**,** a traditional Japanese herbal medicine (Kampo) (n=26)<br>- Contains 12 crude Japanese herbs: Rehmannia root, Angelica root, Atractylodes rhizome, Poria Sclerotium, Ginseng, Cinnamon bark, Polygala root, Peony root, Citrus Unshiu peel, Atsragalus	Standard care (n=26)	**Tool:** NCI-CTCAE<br><br>**Time point:** baseline (prior to CTX) & before each CTX treatment	**PN Incidence:**<br>*At the 8th CTX cycle*<br>**Grade ≥2 PN:**<br>- IG: n=2/20 (10%), CG: n=11/20 (55%), **p<0.01** between groups<br>**Grade 1 PN:**<br>- IG: n=18/20 (90%), CG: n=9/20 (45%), p NR<br>- *Asymptomatic cases within Grade 1 classification*: IG: n=6/18 (33%), CG: n=2/9 (22%), p=0.676 between groups<br>**Grade 2 PN:**	**Quality of life:** NR<br><br>**Pain:** NR<br><br>**Physical function:** NR<br><br>**Sleep/fatigue:**<br>*According to the NCI-CTCAE; caused dose reduction or stoppage of oxaliplatin*<br>**Insomnia:**<br>- Any grade: IG: n=0/26 (0%), CG: n=1/26 (4%; grade 2), p=0.49<br>**General malaise:**<br>- Any grade: IG: n=1/26 (4%; grade 2), CG: n=4/26 (15%; grade 2), p=0.19	"Ninjin'yoeito has prophylactic efficacy against oxaliplatin-induced cumulative PN in patients with colorectal cancers and receiving capecitabine/ oxaliplatin

**Setting**: Outpatient<br><br>**Aim:** to assess the efficacy and safety of ninjin'yoeito for preventing CIPN<br><br>**Trial registration:** UMIN (000012745) (HOPE-2 Trial, Phase II)	**Females**: 40%<br><br>**CIPN history:** Ineligible if had PN from any cause.	**Metastases:** NR	**Previous CTX:** no, excluded if had prior CTX	root, Glycyrrhiza, Schisandra fruit<br><br>**Regimen:** total of 9.0 g/day orally with water (divided into 2 or 3 times/day)<br><br>**Duration:** 8 CTX cycles, from day 1 of Cycle 1<br><br>**Compliance:**<br>- IG: 100% in completed cases (n=20)			- IG: n=2/20 (10%), CG: n=8/20 (40%), p NR<br>**Grade 3 PN:**<br>- IG: n=0/20 (0%), CG: n=4/20 (20%), p NR<br>**Any grade that caused dose reduction or stoppage of oxaliplatin:**<br>- IG: n=2/26 (8%; grade 2), CG: n=10/26 (38%; grade 2 (n=8), grade 3 (n=2)), p NR<br><br>**Time to PN:**<br>*Median time to development of grade 2 or 3 PN*<br>- IG: 21.0 weeks (n=2/20), CG: 16.5 weeks (n=11/20), p=0.470 between groups	**Gastrointestinal symptoms:**<br>*According to the NCI-CTCAE; caused dose reduction or stoppage of oxaliplatin*<br>**Nausea & vomiting:**<br>- Any grade: IG: n=0/26 (0%), CG: n=3/26 (12%; grade 2), p=0.18<br>**Anorexia:**<br>- Any grade: IG: n=2/26 (8%; grade 2), CG: n=7/26 (27%; grade 2 (n=3), grade 3 (n=4)), p=0.08<br><br>**Nutrition status:** NR<br><br>**Psychological outcomes:** NR<br><br>**Social outcomes:** NR<br><br>**Treatment outcomes:**<br>**Relative dose intensity of oxaliplatin:**<br>- IG: 83.3 ± 3.3%, CG: 72.3 ± 3.3%, **p=0.02** between groups<br>**Incidence of 100% relative dose intensity of oxaliplatin:**<br>- IG: n=8/20 (40%), CG: n=1/20 (5%), **p<0.05** between groups<br>**Relative dose intensity of capecitabine:**<br>- IG: 90.5 ± 2.4% CG: 78.4 ± 3.1%, **p<0.01** between groups<br>**Recurrence-free survival rate:**<br>- At 60 months: IG: 0.65, CG: 0.55 (values estimated from graph); log-rank test: p=0.228<br>**Overall survival rate:**<br>- At 60 months: IG: 0.85, CG: 0.80 (values estimated from graph); log-rank test: p=0.574<br>**Completion rate of 8-week CTX course:**<br>- IG: n=20/26 (77%), CG: n=20/26 (77%)<br>- Reasons for discontinued CTX: adverse reactions (cumulative PN, anorexia, nausea, general malaise, neutropenia, thrombocytopenia, and insomnia in both groups; IG: n=4, CG: n=4), recurrence (IG: n=1, CG: n=1), patients' decline (IG: n=1, CG: n=0), cerebrovascular event (IG: n=0, CG: n=1)<br><br>**Financial:** NR<br><br>**Adverse events:**<br>**Hypokalemia and leg edema relatable to intervention:**<br>- IG: n=1/26 (4%), CG: n=0/26 (0%), p=0.49<br>**Neutropenia:**<br>*According to the NCI-CTCAE; caused dose reduction or stoppage of oxaliplatin*<br>- Any grade: IG: n=3/26 (12%; grade 3 (n=2), grade 4 (n=1)), CG: n=5/26 (19%; grade 3 (n=3), grade 4 (n=2)), p=0.45<br>**Thrombocytopenia:**<br>*According to the NCI-CTCAE; caused dose reduction or stoppage of oxaliplatin*<br>- Any grade: IG: n=4/26 (15%; grade 1 (n=3, grade 2 (n=1)), CG: n=1/26 (4%; grade 2), p=0.19	regimen as postoperative adjuvant chemotherapy. NYT can contribute to the maintenance of the standard of doses of capecitabine/ oxaliplatin regimen."

Niskioka et al., 2011<br><br>**Country:** Japan<br><br>**Recruitment dates:** Jan 2007-Dec 2009<br><br>**Setting:** Outpatient<br><br>**Aim:** Efficacy of Goshajinkigan for oxaliplatin-induced PN<br><br>**Trial registration:** UMIN (000002494).	**N:** 45<br><br>**Attrition:** 0%<br><br>**Age (yrs):** Adults: Median (range):<br>- IG: 67 (48-77)<br>- CG: 65 (52-80)<br><br>**Females:** 51%<br><br>**CIPN history:** Ineligible if had PN i.e. focused on CIPN prevention	**Type:** Colorectal<br>- Colon (69%)<br>- Rectum (31%)<br><br>**Advanced Cancer Definition:** Advanced non-resectable or recurrent, metastatic<br><br>**Metastases:**<br>- Liver (53%)<br>- Lung (16%)<br>- Local (9%)<br>- Lymph node (11%)<br>- Other (11%)	**Type**: oxaliplatin (85 mg/m2) &<br>5-FU (2400mg/m2 46 hr infusion).<br>If had grade 3 neuropathy, oxaliplatin dose reduced to 75% of previous dose.<br><br>**Frequency:** Once every two weeks<br><br>**Duration:** One course of CTX. Median (range) CTX cycles received:<br>- IG: 13 (4-32)<br>- CG: 12 (4-28)<br><br>**Ongoing or completed CTX:** Ongoing<br><br>**Previous CTX:** Yes (18%)	**Strategy:** Goshajinkigan, a traditional Japanese herbal medicine (Kampo) (n=22)<br>- Contains 10 crude Japanese herbs: Rehmannia root, Achyranthes root, Cornus fruit, Dioscorea rhizome, Plantago seed, Alisma Rhizome, Poria Sclerotium, Moutan bark, Cinnamon bark, and aconite root<br><br>**Regimen:** 7.5g/day divided into 2-3 doses, orally, before meals or between meals.<br><br>**Duration:** NR; One course of CTX<br><br>**Compliance:** 100%	Standard care (n=23)	**Tool:** DEB-NTC<br><br>**Time point:** Baseline (prior to CTX) & before each CTX treatment	**Incidence of PN**<br>**Incidence of grade 3 PN:**<br>- *After 5 CTX cycles:* IG: 0%, CG: 0%<br>- *After 10 CTX cycles:* IG: 0%, CG: 12%<br>- *After 15 CTX cycles:* IG: 0%, CG: 55%<br>- *After 20 CTX cycles:* IG: 33%, CG: 75%<br>- Sig lower in IG than CG, **p<0.01**, log-rank test<br><br>**Incidence of grade 2 and 3 PN:**<br>- *After 5 CTX cycles:* IG: 10%, CG: 10%<br>- *After 10 CTX cycles:* IG: 0%, CG: 48%<br>- *After 15 CTX cycles:* IG: 40%, CG: 88%<br>- *After 20 CTX cycles:* IG: 65%, CG: 100%<br>- p>0.05 between groups<br><br>**Incidence of grade 1 or worse PN**:<br>- p>0.05 between groups (data NR)	**Quality of life:** NR<br><br>**Pain:** NR<br><br>**Physical function:** NR<br><br>**Sleep/fatigue:** NR<br><br>**Gastrointestinal symptoms:**<br>*Grade 3 adverse events as measured by the NCI-CTC:*<br>- Nausea: IG: n=4 (18%), CG: n=2 (9%), p=0.34<br>- Vomiting: IG: n=1 (5%), CG: n=1 (4%), p=0.97<br>- Diarrhea: IG: n=2 (9%), CG: n=2 (9%), p=0.41<br>- Mucositis: IG: n=2 (9%), CG: n=2 (9%), p=0.96<br>- Anorexia: IG: n=0 (0%), CG: n=1 (4%), p=0.32<br><br>**Nutrition status:** NR<br><br>**Psychological outcomes:** NR<br><br>**Social outcomes:** NR<br><br>**Treatment outcomes:**<br>**Anti-tumour effect of CTX:**<br>*Tumour response to mFOLFOX6 (measured by RECIST):*<br>- Response rate (complete/partial response): IG: n=15 (68%), CG: n=13 (57%), p=0.62<br>- Disease control rate (complete/partial response & stable disease): IG: n=20 (91%), CG: n=21 (92%), p=0.96<br>- Complete response: IG: n=0 (0%), CG: n=0 (0%)<br>- Partial response: IG: n=15 (68%), CG: n=13 (57%)<br>- Stable disease: IG: n=5 (23%), CG: n=8 (35%)<br>- Progressive disease: IG: n=2 (9%), CG: n=2 (8%)<br>**CTX discontinuation:**<br>*Due to:*<br>- Progressive disease: IG: n=0 (0%), CG: n=9 (39%)<br>- Allergy to oxaliplatin: IG: n=0 (0%), CG: n=1 (4%)<br>- Persistent grade 3 peripheral neuropathy: IG: n=0 (0%), CG: n=1 (4%)<br><br>**Financial:** NR<br><br>**Adverse events:**<br>*Grade 3 adverse events as measured by the NCI-CTC:*<br>- Neutropenia: IG: n=3 (14%), CG: n=1 (4%), p=0.27<br>- All grade 3 toxicity (except PN): IG: n=8 (36%), CG: n=8 (35%), p=0.84	**Author conclusions:** "The Kampo medicine, Goshajinkigan, safely reduced the incidence of severe neuropathy by mFOLFOX6 regimen without any adverse influence on the response rate to mFOLFOX6. Therefore, Goshajinkigan is useful in preventing oxaliplatin-induced neuropathy in patients with non-resectable or recurrent colorectal cancer."
Oki et al., 2015<br><br>**Country:** Japan	**N:** 186<br><br>**Attrition:** 2%	**Type:** Colorectal<br><br>**Advanced Cancer**	**Type**: oxaliplatin (85 mg/m2) &<br>5-FU (2400mg/m2 46 hr infusion).	**Strategy:** Goshajinkigan, a traditional Japanese herbal medicine (Kampo) (n=93)	Placebo (n=93)	**Tool:** NCI CTCAE & DEB-NTC	**PN incidence:**[a]<br>*According to the NCI-CTCAE*<br>**Grade ≥1 PN:**<br>- IG: n=84 (94%), CG: n=87 (94%), p=0.42	**Quality of life:** NR<br><br>**Pain:** NR<br><br>**Physical function:** NR	"Goshajinkigan did not prevent oxaliplatin-associated

**Recruitment dates:** NR<br><br>**Setting:** Outpatient<br><br>**Aim:** Efficacy of Goshajinkigan for CIPN<br><br>**Trial registration:** UMIN (000004282) (GENIUS Trial; Phase II)	**Age (yrs):**<br>Adults:<br>- IG: 62.4 ± 10.6<br>- CG: 60.4 ± 11.5<br><br>**Females:** 45%<br><br>**CIPN history:** Ineligible if had PN of any grade	**Definition:** Stage III (Japanese Classification of Colorectal Carcinoma)<br><br>**Metastases:**<br>- Lymph node N1 (68%)<br>- Lymph node N2/ N3 (30%)	Oxaliplatin dose modifications/ skipping were not allowed for patients who had grade 1 PN. Dose reduction of oxaliplatin to 75 mg/ m2 was allowed for patients who had persistent grade 2 PN.<br><br>**Frequency:** Once every two weeks<br><br>**Duration:** One course of CTX of 12 CTX cycles<br><br>**Ongoing or completed CTX:** Ongoing<br><br>**Previous CTX:** NR	- Contains 10 crude Japanese herbs: Rehmannia root, Achyranthes root, Cornus fruit, Dioscorea rhizome, Plantago seed, Alisma Rhizome, Poria Sclerotium, Moutan bark, Cinnamon bark, and Aconite root<br><br>**Regimen:** 7.5g/day orally before or between meals; frequency NR<br><br>**Duration:** One course of CTX of 12 CTX cycles (6 months), starting on the first day of CTX<br><br>**Compliance:** NR		**Time point:** NR; Figures suggest baseline (prior to CTX) & before each CTX treatment	**Grade ≥2 PN:**<br>- IG: n=45 (51%), CG: n=29 (31%), **p=0.02**<br>**Grade 0 PN:**<br>- IG: n=5 (6%), CG: n=6 (7%), p=0.76<br>**Grade 1 PN:**<br>- IG: n=39 (44%), CG: n=58 (62%), **p=0.006**<br>**Grade 2 PN:**<br>- IG: n=30 (34%), CG: n=19 (20%), p=0.007<br>**Grade 3 PN:**<br>- IG: n=15 (17%), CG: n=10 (11%), p=0.29<br><br>**Time to PN:**<br>**Grade ≥2 PN:**<br>- HR for time to PN: 1.908, 95% CI: 1.181-3.083, **p=0.007** (time to PN sig less in IG group)<br>**Grade ≥1 PN:**<br>- HR for time to PN: 1.450, 95% CI: 1.029-2.042, p=0.051<br>**Subgroup analyses:**<br>- Male (n=99): HR: 1.672, 95% CI: 0.894-3.127, p=0.108<br>- Female (n=83): HR: 1.976, 95% CI: 0.971-4.021, p=0.060<br>- <65 years old (n=106): HR: 1.693, 95% CI: 0.914-3.136), p=0.094<br>- ≥65 years old (n=76): HR: 1.865, 95% CI: 0.908-3.833, p=0.090<br>- pStage IIIa (n=128): HR: 2.158, 95% CI: 1.244-3.743, **p=0.006**<br>- pStage IIIb (n=54): HR: 1.058, 95% CI: 0.429-2.611, p=0.902<br>- Tumour location: right (n=61): HR: 1.900, 95% CI: 0.813-4.441, p=0.139<br>- Tumour location: left (n=121): HR: 1.707, 95% CI: 0.971-3.000, p=0.063<br>- Ccr <60mL/min (n=21): HR: 5.152, 95% CI: 0.616-43.104, p=0.130<br>- Ccr ≥60mL/min (n=161): HR: 1.657, 95% CI: 1.019-2.695, **p=0.042**	**Sleep/fatigue:**<br>**Fatigue:**<br>*According to the NCI-CTCAE*<br>- Any grade: IG: n=59 (66%), CG: n=62 (67%), p=0.877<br>- Grade 3/4: IG: n=0 (0%), CG: n=0 (0%)<br><br>**Gastrointestinal symptoms:**<br>*According to the NCI-CTCAE*<br>**Nausea:**<br>- Any grade: IG: n=65 (72%), CG: n=71 (76%), p=0.612<br>- Grade 3/4: IG: n=2 (2%), CG: n=2 (2%), p=1.000<br>**Vomiting:**<br>- Any grade: IG: n=23 (26%), CG: n=31 (33%), p=0.261<br>- Grade 3/4: IG: n=1 (1%), CG: n=0 (0%), p=0.492<br>**Diarrhea:**<br>- Any grade: IG: n=32 (36%), CG: n=28 (30%), p=0.529<br>- Grade 3/4: IG: n=3 (3%), CG: n=2 (2%), p=0.679<br>**Anorexia:**<br>- Any grade: IG: n=62 (69%), CG: n=68 (73%), p=0.625<br>- Grade 3/4: IG: n=3 (3%), CG: n=3 (3%), p=1.000<br><br>**Nutrition status:** NR<br><br>**Psychological outcomes:** NR<br><br>**Social outcomes:** NR<br><br>**Treatment outcomes:**<br>**Oxaliplatin dose intensity until the onset of grade ≥2 PN:**<br>- Relative dose intensity planned protocol (%): IG: 94.72 ± 7.05; CG: 94.98 ± 7.16; p=0.803<br>- Dose intensity (mg/m^2/cycle): IG: 70.90 ± 10.17; CG: 67.14 ± 12.90; **p=0.033**<br>- Relative dose intensity (%): IG: 83.41 ± 11.96; CG: 78.99 ± 15.17; **p=0.033**<br>**Mean number of cycles completed:**<br>- IG: 9.0; CG: 8.3; p NR<br><br>**Financial:** NR<br><br>**Adverse events:**<br>*According to the NCI-CTCAE*<br>**Allergic reaction:**<br>- Any grade: IG: n=15 (17%), CG: n=17 (18%), p=0.847<br>- Grade 3/4: IG: n=1 (1%), CG: n=2 (2%), p=1.000<br>**Chromatosis:**<br>- Any grade: IG: n=19 (21%), CG: n=17 (18%), p=0.711<br>- Grade 3/4: IG: n=0 (0%), CG: n=0 (0%)<br>**Anemia:**<br>- Any grade: IG: n=54 (60%), CG: n=52 (56%), p=0.654<br>- Grade 3/4: IG: n=0 (0%), CG: n=1 (1%), p=1.000<br>**Leucopoenia:**	peripheral neuropathy. Goshajinkigan may prevent acute mild neuropathy and increase the dose intensity of oxaliplatin, but it may consequently cause severe neuropathy. At present, herbal medicine (Kampo) should not be used to reduce acute mild neuropathy because they may induce chronic severe neuropathy."

							- No comorbidities (n=129): HR: 1.748, 95% CI: 1.009-3.026, **p=0.046**<br>- Comorbidity (n=53): HR: 1.917, 95% CI: 0.749-4.905, p=0.174	- Any grade: IG: n=55 (61%), CG: n=59 (63%), p=0.764<br>- Grade 3/4: IG: n=5 (6%), CG: n=6 (7%), p=1.000<br>**Neutropenia:**<br>- Any grade: IG: n=63 (70%), CG: n=70 (75%), p=0.507<br>- Grade 3/4: IG: n=32 (36%), CG: n=39 (42%), p=0.448<br>**Thrombocytopenia:**<br>- Any grade: IG: n=55 (61%), CG: n=47 (51%), p=0.181<br>- Grade 3/4: IG: n=2 (2%), CG: n=2 (2%), p=1.000	
**Technology-facilitated symptom self-management**									
Given et al., 2008<br><br>**Country:** USA<br><br>**Recruitment dates:** NR<br><br>**Setting:** Outpatient, in-home<br><br>**Aim:** To assess an automated telephone symptom management for symptom response in breast cancer, compared to a nurse-led cognitive behavioral intervention.	**N:** 47<br><br>**Attrition:** NR<br><br>**Age (yrs):** Adults ≥21<br><br>**Females:** 100%<br><br>**CIPN history:** NR	**Type:** Breast<br><br>**Advanced Cancer Definition:** Metastatic<br><br>**Metastases:** present in 100%	**Type:** Mixed<br><br>**Frequency:** NR<br><br>**Duration:** NR<br><br>**Ongoing or completed CTX:** Ongoing<br><br>**Previous CTX:** NR	**Strategy:** Automated educational telephone symptom management (n=24)<br>- Pre-recorded voice asks patients about severity of 15 symptoms (including PN), for each rated ≥4 patients were asked to read that part of the symptom management guide and at the next call were asked whether they tried the strategies and if they were helpful.<br><br>**Regimen:** weekly phone calls for 4 weeks, then at 6 weeks and 8 weeks<br><br>**Duration:** 8 weeks<br><br>**Compliance:** NR	Nurse-administered symptom management (n=23); followed cognitive behavior model.	**Tool:** 11-point Likert scale, from 0 (absence), to 10 (worst severity)<br><br>**Time point:** baseline (pre-intervention) and 10- and 16-weeks post-baseline	**Peripheral neuropathy symptom response:**<br>*Median time in days to symptom response:*<br>- IG: 35; CG: >55, p NR	**Quality of life:** NR<br><br>**Pain:**<br>*Median time in days to symptom response:*<br>- IG: 34; CG: 35, p NR<br><br>**Physical function:**<br>**Weakness:**<br>*Median time in days to symptom response:*<br>- IG: 24; CG: 55, p NR<br><br>**Sleep/fatigue:**<br>**Fatigue:**<br>*Median time in days to symptom response:*<br>- IG: 55; CG: >55, p NR<br>**Sleep disturbance:**<br>*Median time in days to symptom response:*<br>- IG: 14; CG: 18, p NR<br><br>**Gastrointestinal symptoms:**<br>**Appetite loss:**<br>*Median time in days to symptom response:*<br>- IG: 7; CG: 28, p NR<br>**Constipation:**<br>*Median time in days to symptom response:*<br>- IG: 15; CG: 51, p NR<br>**Diarrhea:**<br>*Median time in days to symptom response:*<br>- IG: 7; CG: >53, p NR<br>**Vomiting:**<br>*Median time in days to symptom response:*<br>- IG: 14.0; CG: 10.5, p NR<br>**Dry mouth:**<br>*Median time in days to symptom response:*<br>- IG: 29; CG: 7, p NR<br><br>**Nutrition status:** NR	"An automated voice response system that monitored symptom severity and directed patients with cancer to specific strategies contained in a symptom self-management guide proved more effective than telephone strategies tailored by nurses among patients with metastatic breast cancer."<br><br>*** Only results for metastatic disease extracted and reported here**

**Trial registration:** NR								**Psychological outcomes:**<br>**Anxiety:**<br>*Median time in days to symptom response:*<br>- IG: 14; CG: >22.5, p NR<br>**Depression:**<br>*Median time in days to symptom response:*<br>- IG: 14; CG: 24, p NR<br>**Difficulty remembering:**<br>*Median time in days to symptom response:*<br>- IG: 11; CG: 53, p NR<br><br>**Social outcomes:** NR<br><br>**Treatment outcomes:** NR<br><br>**Financial:** NR<br><br>**Adverse events:** NR	
Kim et al., 2018<br><br>**Country:** Korea<br><br>**Recruitment dates:** **Setting:** Sep 2013-Sep 2014<br><br>**Aim**: to evaluate if patient education using a mobile game may increase drug compliance, decrease physical side effects of chemotherapy, and	**N:** 76<br><br>**Attrition:** 5%<br><br>**Age (yrs):** Adults 18-65: Mean 50.9 ± 7.0<br><br>**Females:** 100%<br><br>**CIPN history:** NR	**Type:** Breast<br><br>**Advanced Cancer Definition:** Stage IV metastatic<br><br>**Metastases:** 100% had metastases	**Type**: Combination of taxanes, anthracyclines, capecitabine, & cisplatin<br><br>**Frequency:** NR<br><br>**Duration:** NR<br><br>**Ongoing or completed CTX:** Ongoing<br><br>**Previous CTX:** only eligible if having at least 3rd-line CTX	**Strategy:** Symptom self-management mobile phone game: ILOVEBREAST (n=36)<br>- multiplayer, social network, & platform-based features<br>- Features: education and support for preventing side effects of CTX, encouragement of mood and activity (e.g., exercise, cooking, social games), & self-assessment using a personal avatar<br>- Game quest involves completing activities such as taking medication at right time, cooking a meal.<br>- The game presents an avatar that can prevent side effects of numbness and hair loss by purchasing gloves or hats.<br><br>**Regimen:** Recommended to play game for >30minutes per day, 3 times per week	Standard care: conventional symptoms management education via booklet (n=40)<br>- Advised to read for >30 minutes per day, 3 times per week	**Tool:** CTCAE<br><br>**Time point:** Baseline (pre-intervention) & post-intervention (after 3 weeks)	**Numbness of hand/foot incidence:**<br>- *Any grade:* IG: n=0/34 (0%); CG: n=22/38 (58%), **p=0.02** between groups<br>- *Grade* ≥3: IG: 0/34 (0%); CG: n=3/38 (8%), p=0.28 between groups	**Quality of life:**<br>*Mean score (measured by World Health Organization Quality of Life-BREF Scale; higher scores indicate higher quality of life):*<br>**Quality of life:**<br>- Baseline: IG: 77.5 ± 3.4; CG: 76.8 ± 4.5<br>- Post-intervention: IG: 74.9 ± 3.5; CG: 72.2 ± 5.3<br>- p between groups: **p=0.01**<br>**Overall quality:**<br>- Baseline: IG: 2.3 ± 0.7; CG: 2.3 ± 0.6<br>- Post-intervention: IG: 2.5 ± 0.7; CG: 2.5 ± 0.6<br>- p between groups: p=0.65<br>**Overall health:**<br>- Baseline: IG:1.9 ± 0.5; CG: 1.7 ± 0.6<br>- Post-intervention: IG: 2.1 ± 0.7; CG: 2.3 ± 0.6<br>- p between groups: p=0.08<br><br>**Pain:** NR<br><br>**Physical function:**<br>**Physical health:**<br>*Mean score (measured by World Health Organization Quality of Life-BREF Scale; higher scores indicate better health):*<br>- Baseline: 19.1 ± 2.2; CG: 18.7 ± 1.8<br>- Post-intervention: IG: 20.4 ± 2.2; CG: 21.1 ± 1.7<br>- p between groups: **p=0.003**<br><br>**Sleep/fatigue:**<br>**Fatigue:**<br>*Incidence of adverse event measured by CTCAE:*<br>- *Any grade:* IG: n=16/34 (47%); CG: n=29/38 (76%), **p=0.02** between groups<br>- *Grade* ≥3: IG: 1/34 (3%); CG: n=12/38 (32%), **p=0.002** between groups	"ILOVEBREAST, was helpful in educating adult patients with breast cancer receiving cytotoxic chemotherapy. The game was associated with improved drug compliance, decreased prevalence rates of physical side effects, and better QoL. Patient education with smartphone mobile games can be used as an easy, fun, and effective measure to promote treatment adherence, which may potentially lead to

improve psychological status in breast cancer patients<br><br>**<u>Trial registration:</u>** Clinical Trials (NCT03205969)				**<u>Duration:</u>** 3 weeks<br><br>**<u>Compliance:</u>** patients used about 40% of the game contents.				**<u>Gastrointestinal symptoms:</u>**<br>*Incidence of adverse event measured by CTCAE:*<br>**Nausea:**<br>- *Any grade:* IG: n=29/34 (85%); CG: n=23/38 (61%), **p=0.02** between groups<br>- *Grade* ≥3: IG: 5/34 (15%); CG: n=0/38 (0%), **p=0.02** between groups<br>**Decreased appetite:**<br>*Incidence of adverse event measured by CTCAE:*<br>- *Any grade:* IG: n=16/34 (47%); CG: n=11/38 (29%), p=0.18 between groups<br>- *Grade* ≥3: IG: 3/34 (9%); CG: n=6/38 (16%), p=0.59 between groups<br>**Diarrhea or constipation:**<br>- *Any grade:* IG: n=7/34 (21%); CG: n=9/38 (24%), p=0.97 between groups<br>- *Grade* ≥3: IG: 1/34 (3%); CG: n=5/38 (13%), p=0.25 between groups<br><br>**<u>Nutrition status:</u>** NR<br><br>**<u>Psychological outcomes:</u>**<br>**Psychological health:**<br>*Mean score (measured by World Health Organization Quality of Life-BREF Scale; higher scores indicate better health):*<br>- Baseline: 18.1 ± 1.4; CG: 17.6 ± 43.1<br>- Post-intervention: IG: 18.7 ± 1.4; CG: 19.3 ± 4.3<br>- p between groups: **p=0.02**<br>**Depression:**<br>*Mean score (measured by Beck's Depression Inventory; higher scores indicate greater depression symptoms):*<br>- Baseline: 13.1 ± 3.5; CG: 12.4 ± 5.6<br>- Post-intervention: IG: 15.7 ± 3.7; CG: 14.9 ± 5.2<br>- p between groups: p=0.99<br>**Anxiety:**<br>*Mean score (measured by Spielberger State-Trait Anxiety Scale; higher scores indicate greater anxiety):*<br>- Baseline: 37.4 ± 3.8; CG: 37.9 ± 3.3<br>- Post-intervention: IG: 40.6 ± 3.6; CG: 42.0 ± 3.8<br>- p between groups: p=0.21<br><br>**<u>Social outcomes:</u>**<br>**Social relationships:**<br>*Mean score (measured by World Health Organization Quality of Life-BREF Scale; higher scores indicate better relationships):*<br>- Baseline: 9.3 ± 1.3; CG: 9.6 ± 1.6<br>- Post-intervention: IG: 9.4 ± 1.2; CG: 9.9 ± 1.9<br>- p between groups: p=0.67<br><br>**<u>Treatment outcomes:</u>**<br>**Medication compliance:**	improved survival."

								Mean score (measured by Korean Medication Adherence Rating Scale; higher scores indicate better adherence):<br>- IG: 7.6 ± 0.7; CG: 6.5 ± 0.5, **p<0.001** between groups<br><br>**Financial:** NR<br><br>**Adverse events:**<br>*Incidence of adverse event measured by CTCAE:*<br>- *Stomatitis:* IG: n=0/34 (0%); CG: n=4/38 (11%), p=0.15 between groups<br>- *Hair loss:* IG: n=0/34 (0%); CG: n=10/38 (26%), p=0.27 between groups	

[a] p value reported where not measured in study.

5FU: Fluorouracil; C: chemotherapy cycle; CG: control group; CIPN: chemotherapy-induced peripheral neuropathy; CTX: chemotherapy; CMAP: compound muscle action potential; D: day; DEB-NTC: Neurotoxicity Criteria of Debiopharm; EORTC QLQ-C30: European Organization for Research and Treatment of Cancer Quality of Life Questionnaire; EORTC QLQ-LC13: European Organization for Research and Treatment of Cancer Quality of Life Questionnaire Lung Cancer 13; EPA: eicosapentaenoic acid; FACT-G: Functional Assessment of Cancer Therapy - General; FACT/GOG-NTX: Functional Assessment of Cancer Therapy Gynecologic Oncology Group Neurotoxicity; hr: hour; IG: intervention group; min: minutes; mBPSPN: Modified Balis Pediatric Scale of Peripheral Neuropathies; NCI-CTCAE: National Cancer Institute Common Terminology Criteria for Adverse Events (NCI-CTCAE PN Criteria: Grade 1 (no or mild numbness), grade 2 (moderate numbness without influence on activities of daily living (ADL)), grade 3 (severe numbness interfering with ADL), grade 4 (permanent sensory losses that are disabling)); NCV: nerve conduction velocity; NSCL: Non-small cell lung cancer; NR: not reported; PN: peripheral neuropathy; RECIST: Guidelines for Evaluation of the Response to Treatment in Solid Tumors; SAP: sensory amplitude potential; TOI of FACT/GOG-NTX: Trial Outcome Index of Functional Assessment of Cancer Therapy Gynecologic Oncology Group Neurotoxicity; UK: United Kingdom; USA: United States of America; wk: week; yrs: years.

Supplementary File S3. Forest plots of non-significant meta-analyses

Grade 1 CIPN

Study or Subgroup	Experimental Events	Experimental Total	Control Events	Control Total	Weight	Odds Ratio M-H, Random, 95% CI
Motoo 2020	18	20	9	20	46.9%	11.00 [2.00, 60.57]
Oki 2015	39	93	58	93	53.1%	0.44 [0.24, 0.78]
**Total (95% CI)**		**113**		**113**	**100.0%**	**1.98 [0.08, 48.14]**
Total events	57		67			

Heterogeneity: $Tau^2 = 4.90$; $Chi^2 = 12.56$, df = 1 (P = 0.0004); $I^2 = 92\%$
Test for overall effect: Z = 0.42 (P = 0.68)

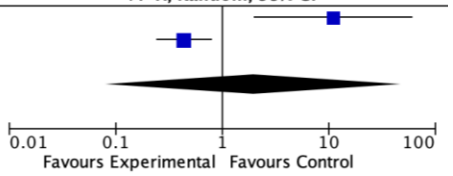

Grade 2 CIPN

Study or Subgroup	Experimental Events	Experimental Total	Control Events	Control Total	Weight	Odds Ratio M-H, Random, 95% CI
Motoo 2020	2	20	8	20	44.5%	0.17 [0.03, 0.92]
Oki 2015	30	93	19	93	55.5%	1.85 [0.95, 3.61]
**Total (95% CI)**		**113**		**113**	**100.0%**	**0.64 [0.06, 6.71]**
Total events	32		27			

Heterogeneity: $Tau^2 = 2.49$; $Chi^2 = 6.67$, df = 1 (P = 0.010); $I^2 = 85\%$
Test for overall effect: Z = 0.38 (P = 0.71)

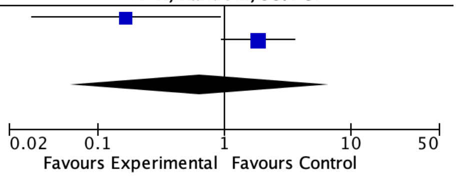

Grade 3 CIPN

Study or Subgroup	Experimental Events	Experimental Total	Control Events	Control Total	Weight	Odds Ratio M-H, Random, 95% CI
Motoo 2020	0	20	4	20	21.5%	0.09 [0.00, 1.78]
Niskioka 2011	7	22	17	23	37.3%	0.16 [0.05, 0.60]
Oki 2015	15	93	10	93	41.2%	1.60 [0.68, 3.76]
**Total (95% CI)**		**135**		**136**	**100.0%**	**0.37 [0.05, 2.52]**
Total events	22		31			

Heterogeneity: $Tau^2 = 2.15$; $Chi^2 = 10.24$, df = 2 (P = 0.006); $I^2 = 80\%$
Test for overall effect: Z = 1.02 (P = 0.31)

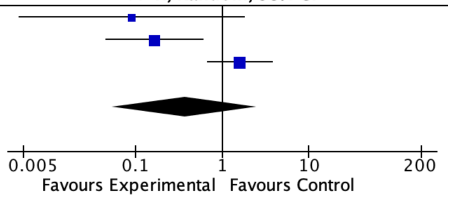

Grade ≥2 CIPN

Study or Subgroup	Experimental Events	Experimental Total	Control Events	Control Total	Weight	Odds Ratio M-H, Random, 95% CI
Motoo 2020	2	20	11	20	34.2%	0.09 [0.02, 0.50]
Niskioka 2011	14	22	23	23	27.4%	0.04 [0.00, 0.68]
Oki 2015	45	93	29	93	38.4%	2.07 [1.14, 3.76]
**Total (95% CI)**		**135**		**136**	**100.0%**	**0.23 [0.01, 3.89]**
Total events	61		63			

Heterogeneity: $Tau^2 = 5.25$; $Chi^2 = 18.44$, df = 2 (P < 0.0001); $I^2 = 89\%$
Test for overall effect: Z = 1.01 (P = 0.31)

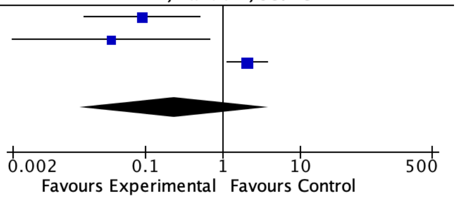

Supplementary Figure S1. Japanese herbal medicine had no significant association with likelihood of CIPN of Grade 1, 2, 3 or ≥2 (n=2-3 studies; n=226-271 participants; GRADE level: very low). Sensitivity analysis according to type of herbal supplement (Goshajinkigan vs. ninjin'yoeito) did not result in significant findings.

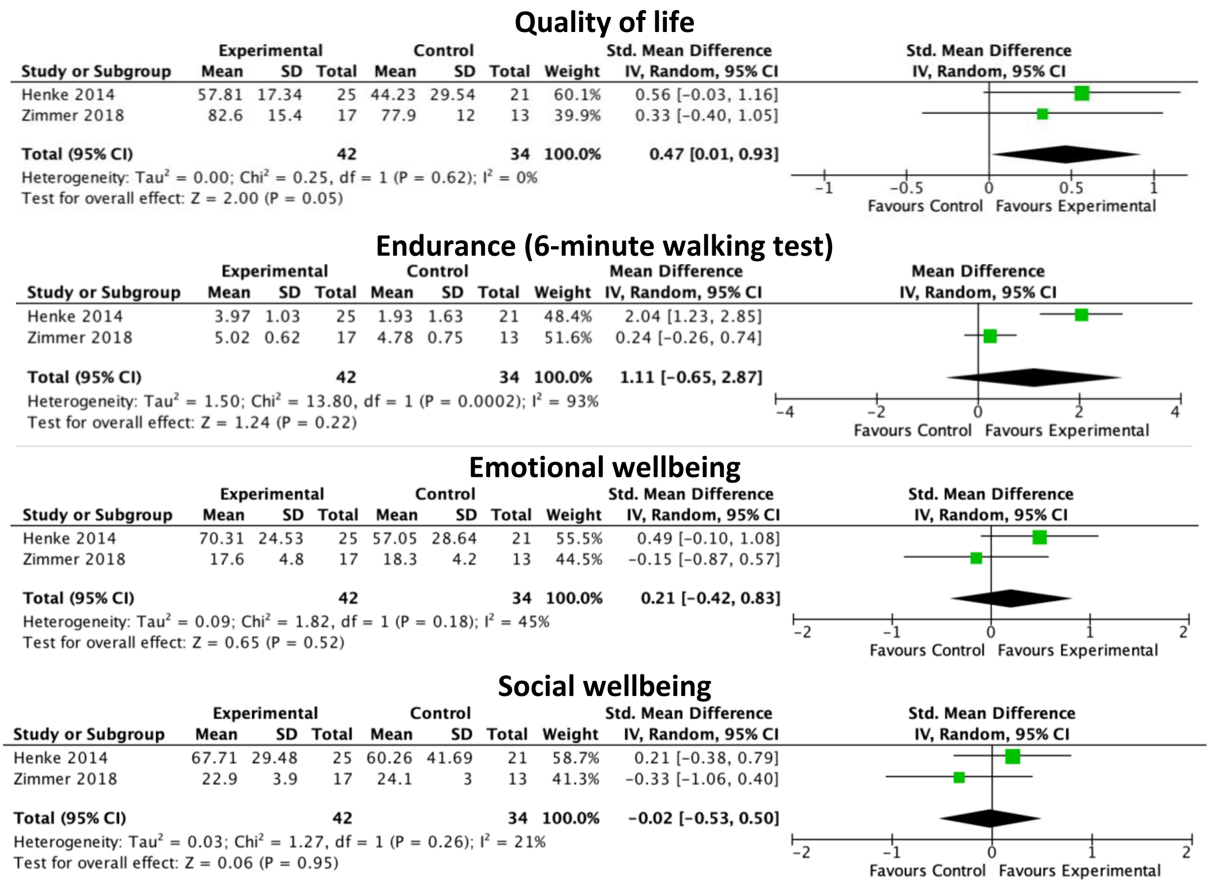

Supplementary Figure S2. Physical exercise had no significant effect on quality of life, endurance, and emotional or social wellbeing (n=2 studies; n=76 participants; GRADE level: very low).

Fatigue

Study or Subgroup	Experimental Events	Experimental Total	Control Events	Control Total	Weight	Odds Ratio M-H, Random, 95% CI
Motoo 2020	1	26	7	26	37.1%	0.11 [0.01, 0.96]
Oki 2015	59	93	62	93	62.9%	0.87 [0.47, 1.59]
**Total (95% CI)**		**119**		**119**	**100.0%**	**0.40 [0.06, 2.93]**
Total events	60		69			

Heterogeneity: Tau^2 = 1.54; Chi^2 = 3.31, df = 1 (P = 0.07); I^2 = 70%
Test for overall effect: Z = 0.90 (P = 0.37)

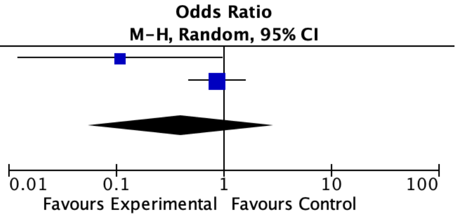

Nausea

Study or Subgroup	Experimental Events	Experimental Total	Control Events	Control Total	Weight	Odds Ratio M-H, Random, 95% CI
Motoo 2020	0	26	3	26	10.4%	0.13 [0.01, 2.58]
Niskioka 2011	4	22	2	23	23.8%	2.33 [0.38, 14.26]
Oki 2015	65	93	71	93	65.8%	0.72 [0.37, 1.38]
**Total (95% CI)**		**141**		**142**	**100.0%**	**0.80 [0.28, 2.23]**
Total events	69		76			

Heterogeneity: Tau^2 = 0.31; Chi^2 = 2.87, df = 2 (P = 0.24); I^2 = 30%
Test for overall effect: Z = 0.44 (P = 0.66)

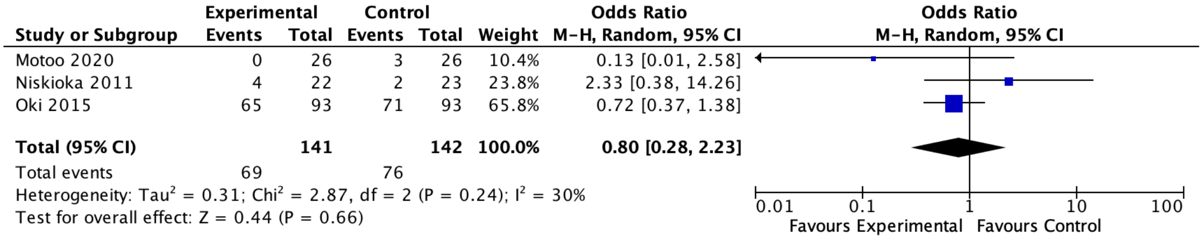

Vomiting

Study or Subgroup	Experimental Events	Experimental Total	Control Events	Control Total	Weight	Odds Ratio M-H, Random, 95% CI
Motoo 2020	0	26	3	26	4.1%	0.13 [0.01, 2.58]
Niskioka 2011	1	22	1	23	4.6%	1.05 [0.06, 17.85]
Oki 2015	23	93	31	93	91.3%	0.66 [0.35, 1.24]
**Total (95% CI)**		**141**		**142**	**100.0%**	**0.63 [0.34, 1.16]**
Total events	24		35			

Heterogeneity: Tau^2 = 0.00; Chi^2 = 1.24, df = 2 (P = 0.54); I^2 = 0%
Test for overall effect: Z = 1.50 (P = 0.13)

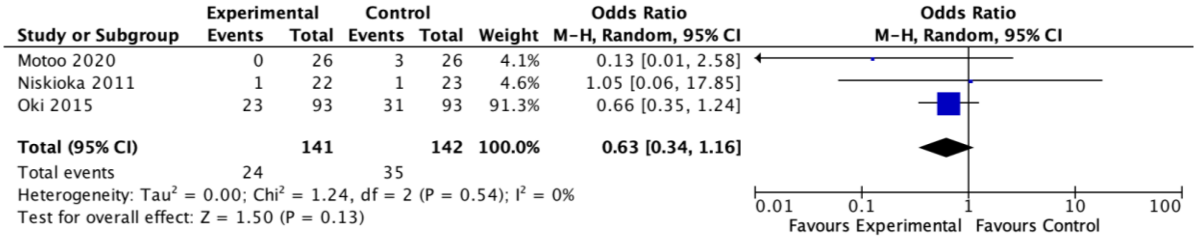

Diarrhea

Study or Subgroup	Experimental Events	Experimental Total	Control Events	Control Total	Weight	Odds Ratio M-H, Random, 95% CI
Niskioka 2011	2	22	2	23	8.3%	1.05 [0.13, 8.18]
Oki 2015	32	93	28	93	91.7%	1.22 [0.66, 2.25]
**Total (95% CI)**		**115**		**116**	**100.0%**	**1.20 [0.67, 2.17]**
Total events	34		30			

Heterogeneity: Tau^2 = 0.00; Chi^2 = 0.02, df = 1 (P = 0.89); I^2 = 0%
Test for overall effect: Z = 0.61 (P = 0.54)

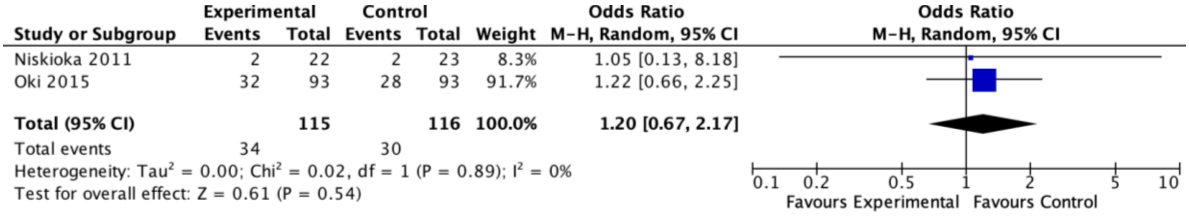

Anorexia

Study or Subgroup	Experimental Events	Experimental Total	Control Events	Control Total	Weight	Odds Ratio M-H, Random, 95% CI
Motoo 2020	2	26	3	26	9.8%	0.64 [0.10, 4.18]
Niskioka 2011	0	22	1	23	3.3%	0.33 [0.01, 8.63]
Oki 2015	62	93	68	93	87.0%	0.74 [0.39, 1.38]
**Total (95% CI)**		**141**		**142**	**100.0%**	**0.71 [0.39, 1.27]**
Total events	64		72			

Heterogeneity: Tau^2 = 0.00; Chi^2 = 0.23, df = 2 (P = 0.89); I^2 = 0%
Test for overall effect: Z = 1.16 (P = 0.25)

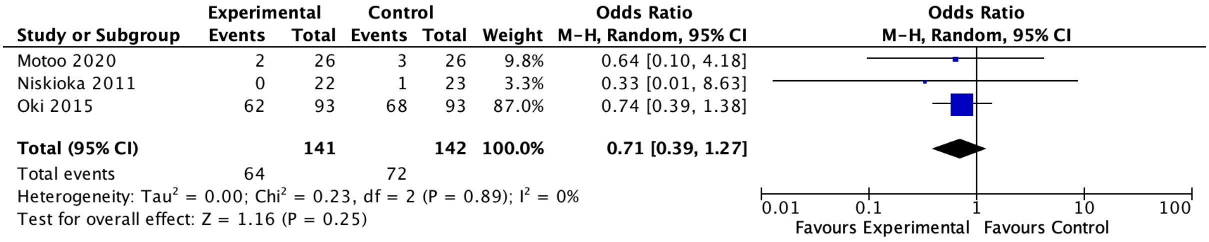

Relative dose reduction of oxaliplatin

Study or Subgroup	Experimental Mean	Experimental SD	Experimental Total	Control Mean	Control SD	Control Total	Weight	Std. Mean Difference IV, Random, 95% CI
Motoo 2020	83.3	3.3	26	72.3	3.3	26	49.0%	3.28 [2.43, 4.14]
Oki 2015	83.41	11.96	93	78.99	15.17	93	51.0%	0.32 [0.03, 0.61]
**Total (95% CI)**			**119**			**119**	**100.0%**	**1.77 [−1.13, 4.68]**

Heterogeneity: Tau^2 = 4.28; Chi^2 = 41.58, df = 1 (P < 0.00001); I^2 = 98%
Test for overall effect: Z = 1.20 (P = 0.23)

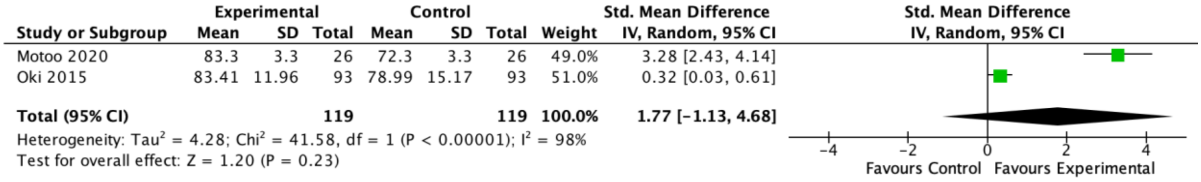

Neutropenia

Study or Subgroup	Experimental Events	Experimental Total	Control Events	Control Total	Weight	Odds Ratio M-H, Random, 95% CI
Motoo 2020	3	26	5	26	13.7%	0.55 [0.12, 2.58]
Niskioka 2011	3	22	1	23	6.0%	3.47 [0.33, 36.24]
Oki 2015	63	93	70	93	80.3%	0.69 [0.36, 1.31]
**Total (95% CI)**		**141**		**142**	**100.0%**	**0.74 [0.41, 1.31]**
Total events	69		76			

Heterogeneity: $Tau^2 = 0.00$; $Chi^2 = 1.87$, df = 2 (P = 0.39); $I^2 = 0\%$
Test for overall effect: Z = 1.04 (P = 0.30)

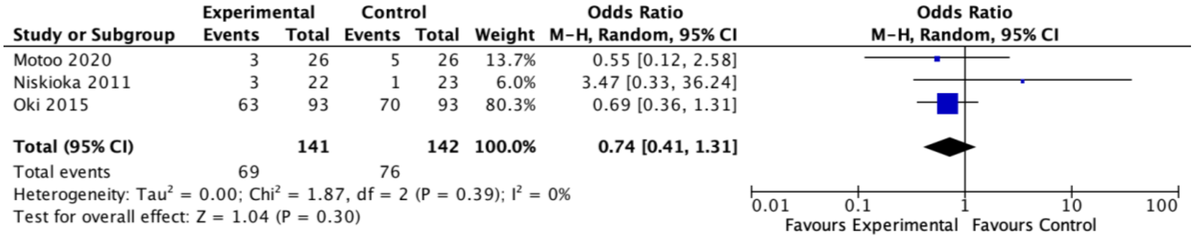

Thrombocytopenia

Study or Subgroup	Experimental Events	Experimental Total	Control Events	Control Total	Weight	Odds Ratio M-H, Random, 95% CI
Motoo 2020	4	26	1	26	6.2%	4.55 [0.47, 43.78]
Oki 2015	55	93	47	93	93.8%	1.42 [0.79, 2.53]
**Total (95% CI)**		**119**		**119**	**100.0%**	**1.52 [0.87, 2.67]**
Total events	59		48			

Heterogeneity: $Tau^2 = 0.00$; $Chi^2 = 0.96$, df = 1 (P = 0.33); $I^2 = 0\%$
Test for overall effect: Z = 1.47 (P = 0.14)

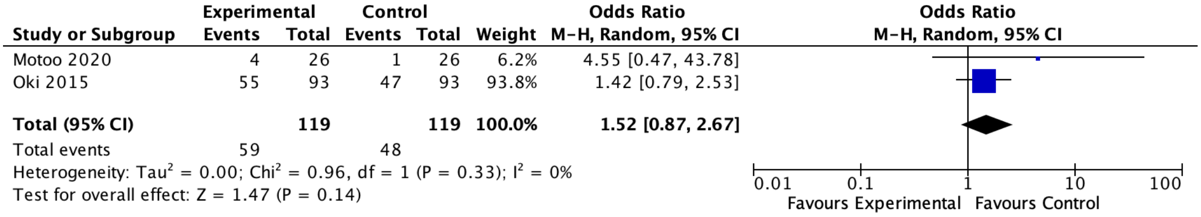

Supplementary Figure S3. Japanese herbal medicine had no significant effect on fatigue, nausea, vomiting, diarrhea, anorexia, relative dose reduction of oxaliplatin, neutropenia, nor thrombocytopenia (n=2-3 studies; n=231-283 participants; GRADE level: very low).

Supplementary File S4. Grading of Recommendations, Assessment, Development and Evaluation (GRADE) Assessment

Supplementary Table S3. Grading of Recommendations, Assessment, Development and Evaluation (GRADE) for each outcome examining the effect of self-administered non-pharmacological interventions on chemotherapy-induced peripheral neuropathy symptoms and related outcomes.

Certainty assessment							Certainty
№ of studies	Study design	Risk of bias	Inconsistency	Indirectness	Imprecision	Other considerations	
**Exercise - Incidence of CIPN**							
1	randomized trial	very serious [e]	not serious	not serious	very serious [a]	none	⨁◯◯◯ VERY LOW
**Exercise - Severity of CIPN**							
2	randomized trials	serious [i]	not serious	not serious	very serious [a]	very strong association [c]	⨁⨁⨁◯ MODERATE
**Exercise - Physical function**							
3	randomized trials	very serious [e]	not serious	not serious	serious [b]	strong association [d]	⨁⨁⨁◯ MODERATE
**Exercise - Endurance**							
2	randomized trials	serious [i]	very serious [f]	not serious	very serious [a]	strong association [d]	⨁◯◯◯ VERY LOW
**Exercise - Quality of life**							
2	randomized trials	serious [i]	not serious	not serious	very serious [a]	none	⨁◯◯◯ VERY LOW
**Exercise – Emotional wellbeing**							
2	randomized trials	serious [i]	not serious	not serious	very serious [a]	none	⨁◯◯◯ VERY LOW
**Exercise – Social wellbeing**							
2	randomized trials	serious [i]	not serious	not serious	very serious [a]	none	⨁◯◯◯ VERY LOW
**Exercise - Pain**							

Certainty assessment							Certainty
№ of studies	Study design	Risk of bias	Inconsistency	Indirectness	Imprecision	Other considerations	
2	randomized trials	very serious [e]	serious [h]	not serious	serious [b]	none	⊕◯◯◯ VERY LOW
**Exercise - Sleep**							
2	randomized trials	very serious [e]	serious [h]	not serious	serious [b]	none	⊕◯◯◯ VERY LOW
**Exercise - Fatigue**							
2	randomized trials	very serious [e]	serious [h]	not serious	serious [b]	none	⊕◯◯◯ VERY LOW
**Exercise - Diarrhea**							
2	randomized trials	very serious [e]	serious [h]	not serious	serious [b]	none	⊕◯◯◯ VERY LOW
**Exercise – Financial problems**							
2	randomized trials	very serious [e]	serious [h]	not serious	serious [b]	none	⊕◯◯◯ VERY LOW
**Exercise – Nutrition status**							
1	randomized trial	very serious [e]	not serious	not serious	very serious [a]	none	⊕◯◯◯ VERY LOW
**Exercise – Lean body mass**							
1	randomized trial	very serious [e]	not serious	not serious	very serious [a]	none	⊕◯◯◯ VERY LOW
**Nutrition supplements – L-glutamine – CIPN incidence**							
1	randomized trial	very serious [e]	not serious	not serious	very serious [a]	none	⊕◯◯◯ VERY LOW
**Nutrition supplements – Curcumin – CIPN incidence**							
1	randomized trial	very serious [e]	not serious	not serious	very serious [a]	none	⊕◯◯◯ VERY LOW
**Nutrition supplements – Omega 3-enriched nutrition drink – CIPN severity**							
1	randomized trial	very serious [e]	not serious	not serious	serious [b]	none	⊕◯◯◯ VERY LOW

Certainty assessment							Certainty
№ of studies	Study design	Risk of bias	Inconsistency	Indirectness	Imprecision	Other considerations	
**Nutrition supplements – L-glutamine – Quality of life**							
1	randomized trial	very serious [e]	not serious	not serious	very serious [a]	none	⨁◯◯◯ VERY LOW
**Nutrition supplements – Omega 3-enriched nutrition drink – Fatigue severity**							
1	randomized trial	very serious [e]	not serious	not serious	serious [b]	none	⨁◯◯◯ VERY LOW
**Nutrition supplements – Omega 3-enriched nutrition drink – Appetite loss**							
1	randomized trial	very serious [e]	not serious	not serious	serious [b]	none	⨁◯◯◯ VERY LOW
**Nutrition supplements – Omega 3-enriched nutrition drink – Body weight maintenance**							
1	randomized trial	very serious [e]	not serious	not serious	serious [b]	none	⨁◯◯◯ VERY LOW
**Nutrition supplements – Curcumin – Body weight maintenance**							
1	randomized trial	very serious [e]	not serious	not serious	very serious [a]	none	⨁◯◯◯ VERY LOW
**Japanese herbal medicine – CIPN incidence Grade 1**							
3	randomized trials	very serious [e]	very serious [f]	not serious	serious [b]	none	⨁◯◯◯ VERY LOW
**Japanese herbal medicine – CIPN incidence Grade 2**							
3	randomized trials	very serious [e]	very serious [f]	not serious	serious [b]	none	⨁◯◯◯ VERY LOW
**Japanese herbal medicine – CIPN incidence Grade 3**							
3	randomized trials	very serious [e]	very serious [f]	not serious	serious [b]	none	⨁◯◯◯ VERY LOW
**Japanese herbal medicine – CIPN incidence Grade 2 & 3**							
3	randomized trials	very serious [e]	very serious [f]	not serious	serious [b]	none	⨁◯◯◯ VERY LOW
**Japanese herbal medicine – Relative dose intensity of oxaliplatin**							

Certainty assessment							Certainty
№ of studies	Study design	Risk of bias	Inconsistency	Indirectness	Imprecision	Other considerations	
2	randomized trials	very serious [e]	very serious [f]	not serious	serious [b]	none	⨁◯◯◯ VERY LOW
**Japanese herbal medicine – Fatigue**							
2	randomized trials	very serious [e]	serious [g]	not serious	serious [b]	none	⨁◯◯◯ VERY LOW
**Japanese herbal medicine – Nausea**							
3	randomized trials	very serious [e]	not serious	not serious	serious [b]	none	⨁◯◯◯ VERY LOW
**Japanese herbal medicine – Vomiting**							
3	randomized trials	very serious [e]	not serious	not serious	serious [b]	none	⨁◯◯◯ VERY LOW
**Japanese herbal medicine – Diarrhea**							
2	randomized trials	very serious [e]	not serious	not serious	serious [b]	none	⨁◯◯◯ VERY LOW
**Japanese herbal medicine – Anorexia**							
3	randomized trials	very serious [e]	not serious	not serious	serious [b]	none	⨁◯◯◯ VERY LOW
**Japanese herbal medicine – Neutropenia**							
3	randomized trials	very serious [e]	not serious	not serious	serious [b]	none	⨁◯◯◯ VERY LOW
**Japanese herbal medicine – Thrombocytopenia**							
2	randomized trials	very serious [e]	not serious	not serious	serious [b]	none	⨁◯◯◯ VERY LOW
**Technology-facilitated symptom self-management – Mobile phone game – CIPN Incidence**							
1	randomized trial	very serious [e]	not serious	not serious	very serious [a]	none	⨁◯◯◯ VERY LOW
**Technology-facilitated symptom self-management – Automated telephone self-management system – Time to CIPN symptom response**							
1	randomized trial	very serious [e]	not serious	not serious	very serious [a]	none	⨁◯◯◯ VERY LOW

Certainty assessment							Certainty
№ of studies	Study design	Risk of bias	Inconsistency	Indirectness	Imprecision	Other considerations	
**Technology-facilitated symptom self-management – Automated telephone self-management system – Time to CIPN symptom response**							
1	randomized trial	very serious [e]	not serious	not serious	very serious [a]	none	⨁◯◯◯ VERY LOW
**Technology-facilitated symptom self-management – Mobile phone game – Quality of life**							
1	randomized trial	serious [i]	not serious	not serious	very serious [a]	none	⨁◯◯◯ VERY LOW
**Technology-facilitated symptom self-management – Mobile phone game – Physical function**							
1	randomized trial	serious [i]	not serious	not serious	very serious [a]	none	⨁◯◯◯ VERY LOW
**Technology-facilitated symptom self-management – Mobile phone game – Nausea**							
1	randomized trial	serious [i]	not serious	not serious	very serious [a]	none	⨁◯◯◯ VERY LOW
**Technology-facilitated symptom self-management – Mobile phone game – Psychological health**							
1	randomized trial	serious [i]	not serious	not serious	very serious [a]	none	⨁◯◯◯ VERY LOW

a. ≤100 participants
b. ≤400 participants
c. Large effect size (SMD >0.8)
d. Moderate effect size (SMD >0.5)
e. Risk of bias found in ‘most’ included studies
f. I^2 50-75%
g. I^2 >75%
h. Difference in effect (i.e., one study significant positive effect, one study non-significant effect)
i. Risk of bias found in ‘some’ included studies
